# *Verrucomicrobium spinosum* essential genome and the divergence of cell division in the PVC superphylum

**DOI:** 10.1016/j.isci.2025.113037

**Published:** 2025-06-30

**Authors:** Valentina Henriques, David Moyano-Palazuelo, Maria Teresa Alonso-Pascual, Manuel Pazos, Damien P. Devos, Elena Rivas-Marin

**Affiliations:** 1 Centro Andaluz de Biología del Desarrollo-CSIC, Pablo de Olavide University, Seville, Spain; 2 Laboratory of Mass Spectrometry (SIdI-UAM), Autonomous University of Madrid, Madrid, Spain; 3 Department of Molecular Biology, Center of Molecular Biology “Severo Ochoa” (UAM-CSIC), Autonomous University of Madrid, Madrid, Spain

**Keywords:** Microbial genomics, Phylogenetics

## Abstract

The *Planctomycetota*-*Chlamydiota*-*Verrucomicrobiota* superphylum comprises bacteria with divergent traits, epitomizing the vast biodiversity of non-model organisms that remains unexplored. Among its members, *Planctomycetota* and *Chlamydiota* exhibit unique adaptations, including variations in peptidoglycan structure and cell division without FtsZ. *Verrucomicrobiota* represents the only name-giving phylum within the PVC not extensively investigated. Here, exhaustive transposon mutagenesis combined with massive sequencing reveal the essential genome of *Verrucomicrobium spinosum*. The essentiality report demonstrates that the *dcw* cluster is essential except *mraW*. Additionally, peptidoglycan analysis evidences that the composition and saccular arrangement are canonical, contrarily to *Planctomycetota* and *Chlamydiota.* Targeted mutagenesis for *V. spinosum* was also developed, thus reinforcing its status as a model for *Verrucomicrobiota*. Ultimately, the evolutionary divergences of the *dcw* cluster in PVC, combined with the previous results, suggest that its Last Common Ancestor was a Gracilicutes-like bacterium dividing by an FtsZ-mediated binary fission with peptidoglycan, from which current phyla diverged.

## Highlights

• TraDIS identified 10% of *V. spinosum* genes as essential
• The *dcw* cluster of *V. spinosum* is essential and its PG is non-acetylated
• The *dcw* cluster distribution suggests FtsZ-dependent division in LPCA
• Targeted mutagenesis established for *V. spinosum*

## Graphical abstract

**Figure:**
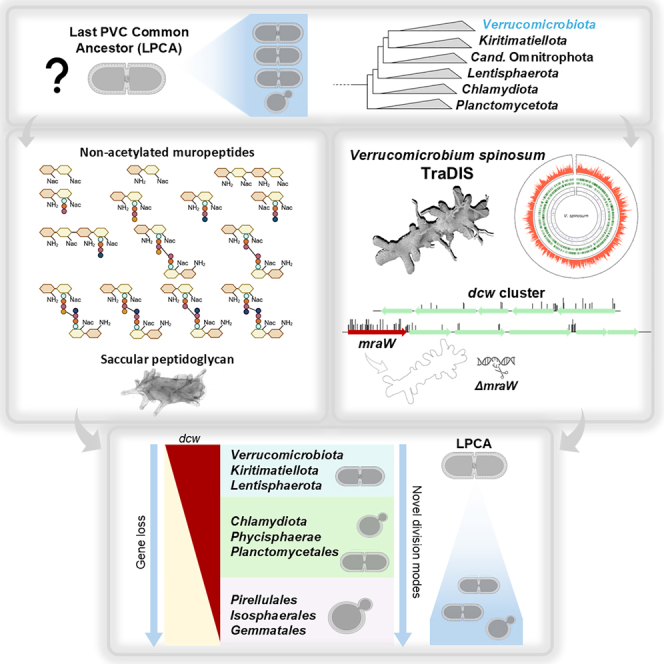

## Introduction

The *Planctomycetota*-*Chlamydiota*-*Verrucomicrobiota* (PVC) superphylum encompasses diverse bacteria with intriguing cellular and molecular biology. These traits remain understudied due to limited model organisms and genetic tools. Members of the phylum inhabit diverse environments, including marine ecosystems,^1^ soils, plant rhizospheres,^2^ and animal guts.^3^ Notably, *Akkermansia muciniphila*, a human gut commensal, is associated with proper development of the host gut and negatively correlated with obesity and diabetes,^4^ while other members of the phylum protect their ciliate symbionts from predators.^5^^,^^6^ Some members inhabit host nuclei,^7^ while others thrive in extreme conditions, such as the methanotrophs that oxidize methane in extreme acidic geothermal ecosystems.^8^ Besides their extensive range of environmental adaptation, they contribute to carbon recycling through carbohydrate-active enzymes and play roles in degrading complex polymers.^9^^,^^10^ They are also known for producing bioactive compounds, including the antimelanoma drug Palmerolide A^11^ and L-DOPA, a Parkinson’s disease treatment.^12^ All of this highlights their metabolic versatility, environmental significance, and potential for therapeutic applications.

Among the many notable features of PVC bacteria, cell division and peptidoglycan (PG) synthesis remain subjects of ongoing debate, as reviewed by Rivas-Marín et al.^13^ While binary fission is the predominant mode of division among most bacteria, members of the PVC superphylum exhibit a remarkable diversity of division strategies, encompassing various forms of budding in addition to conventional binary fission.^14^ Binary fission is a process driven by the divisome, a multiprotein complex regulated by the essential FtsZ protein and linked to the machinery responsible for PG biosynthesis.^15^ Both FtsZ and PG are ubiquitous among bacteria, but exceptions exist, most notably, within certain phyla of the PVC. In particular, FtsZ is absent in *Chlamydiota* and *Planctomycetota*, and their division mechanisms remain poorly understood. Additionally, the presence, nature, and mechanisms of PG synthesis are unclear, as many canonical PG-synthesizing genes are either absent or non-essential.^16^^,^^17^ In contrast *Verrucomicrobiota* retains *ftsZ* and is reported to divide by binary fission.^18^ The occurrence of distinct modes of cell division within the superphylum raises questions regarding the evolutionary divergence in these key cellular processes.^16^^,^^19^^,^^20^ It has been proposed that species diverged, exhibiting variations such as functional modifications of *ftsZ* and other division and cell wall (*dcw)* genes,^21^ as well as their eventual losses.^17^ *Verrucomicrobiota*, however, encodes all *dcw* genes including *ftsZ*, representing supporting evidence for the presence of these genes in the Last PVC Common Ancestor (LPCA).^19^^,^^20^^,^^22^

The canonicity of division and PG synthesis in *Verrucomicrobiota* has been supported by *in silico* identification of *dcw* genes,^18^^,^^19^^,^^20^ a detailed PG description in *A. muciniphila*^23^ and partial in *Verrucomicrobium spinosum* and other verrucomicrobiotas,^24^^,^^25^ as well as functional activity of the genes *murE*,^24^ *murCB* fusion,^26^ and *dapL*^27^ by heterologous complementation. Nonetheless, there has been a lack of essentiality evaluation of the entire *dcw* cluster in *Verrucomicrobiota* under optimal lab conditions. The case of *Planctopirus limnophila*, a budding planctomycetota that lacks *ftsZ* yet encodes the majority of *dcw* genes, of which all but one are non-essential,^17^ illustrates that the presence of a gene in one organism does not necessarily imply its essentiality. In order to explore the essentiality of the *dcw* in *Verrucomicrobiota*, a model organism with fundamental genetic tools is crucial, however, such a model is currently lacking. The phylum *Verrucomicrobiota* is divided into the classes *Methylacidiphilae*, *Opitutia*, *Spartobacteria*, *Terrimicrobiia*, and *Verrucomicrobiia*. We adopt *V*. *spinosum*, from the class *Verrucomicrobiia*, order *Verrucomicrobiales*, for this role, as it is a mesophilic environmental bacterium with relatively quick growth,^28^ outpacing other bacteria in the phylum that require more stringent conditions to grow.

In this study, experimental and computational approaches were combined to investigate the essential gene set of *V*. *spinosum* under optimal laboratory conditions, employing a large-scale transposon mutagenesis technique. Contrarily to *P. limnophila*, essentiality analysis confirmed that most genes within the *dcw* cluster are essential, with the exceptions of *mraW*. To further assess the essentiality of *mraW*, targeted mutagenesis was developed, and growth conditions were optimized, reinforcing the status of *V. spinosum* as a model organism. Additionally, the composition and arrangement of PG were characterized, and together with the *dcw* essentiality, these findings support a classical model of bacterial cell division and peptidoglycan synthesis. Furthermore, we explore potential stages of evolution of the essential gene repertoire in PVC bacteria and trace the evolutionary history of cellular division within the PVC superphylum. Our results provide insights into the divergent molecular and cellular biology of these fascinating bacteria.

## Results and discussion

### Handling optimization of *V. spinosum*

In addition to being the type strain, *V. spinosum* DSM 4136 was chosen due to its phylogenetic position within the PVC superphylum, its relative rapid growth compared to other phylum members, and previous handling reports.^24^^,^^26^^,^^27^^,^^29^ Growth conditions were optimized by supplementing the medium with ammonia and N-acetylglucosamine and by increasing the glucose concentration (Data S1. Excel file containing OD600 values used for graphical representation of Supplementary Figure 1A–1C, Data S2. Excel file containing OD600 values used for graphical representation of Supplementary Figure 1D, Data S3. Excel file containing OD600 values used for graphical representation of Supplementary Figure 1E). Ammonia significantly enhanced the growth, achieving higher cell densities and standardizing its growth pattern (Figure S1). Antibiotic sensitivity tests showed that *V. spinosum* was sensitive to gentamicin (>5 μg.mL^−1^), streptomycin (>5 μg.mL^−1^), ampicillin (>25 μg.mL^−1^), tetracycline (>10 μg.mL^−1^) and kanamycin (>10 μg.mL^−1^), and that it was resistant to chloramphenicol 30 μg.mL^−1^ (Figure S2). In addition to the growth improvement, targeted mutagenesis was also developed (see Method section), positioning *V. spinosum* a as model organism within *Verrucomicrobiota*.

### Saturated mini-Tn*5* library construction and sequencing in *V. spinosum*

TraDIS (Transposon Directed Insertion-site Sequencing) method was employed to create a transposon mutant library in *V. spinosum*. Optimization of the protocol previously reported^29^ was required to enhance efficiency (see Method section). Optimal state of competence is achieved during the initial exponential growth phase, at an OD_600_ of 0.3–0.5. A complex consisting of a mini-Tn*5* transposon bearing a kanamycin resistance cassette together with a transposase was electroporated into competent cells and grown on selective medium. Colonies were pooled directly from plates to construct the library, estimated to be approximately 1.25 million mutants. The occurrence of single transposon insertion was confirmed by whole-genome sequencing of a Tn*5*-mutant. Genomic DNA from the pooled library was sequenced resulting in 8,895,324 sequence reads. After the removal of short reads, poor-quality data, and fragments that did not contain the transposon sequence, 6,137,903 reads were mapped to the *V. spinosum* genome. This resulted in 680,519 unique insertions in the chromosome of which 584,515 insertion events were mapped in coding sequences (CDS), leaving 96,004 events in intergenic regions (Data S4). The insertion sites covered the entire genome length and were evenly distributed; no biases in the transposition events could be detected (Figure S3). The high density of unique insertion sites resulted in an average of one insertion every 12 bp.

### Gene essentiality

We followed a statistical method previously described to determine gene essentiality under lab conditions.^17^^,^^30^ Briefly, the number of unique insertion sites per gene was normalized by gene length. The frequency distribution of the insertion index scores was bimodal, the left mode corresponded to essential genes, and non-essential ones were associated with the right one, while genes in between were deemed as unclear (Figure S4). Two distribution models, gamma and exponential, are fitted to the frequency distribution, and the probability of each gene belonging to one or the other is defined. The ratio of these values defines the log likelihood score. A gene was classified as essential if its log likelihood score was less than log_2_ (12), therefore, if it is 12 times more likely to belong to the essential mode than to the non-essential one. We also used an additional sliding-window protocol to detect insertion-free regions within a gene.^17^

The genome of *V. spinosum* consists of 6,244 genes (including 6,166 CDS, 62 tRNA genes, 12 ribosomal RNA genes, and four others rare RNAs: two tmRNA, one SRP_RNA and one RNase_P_RNA) plus 34 pseudogenes. With this analysis 644 genes were classified as essential (10%), 5,493 as non-essential (88%) and 141 genes (2%) as unclear (Data S5). This list included 15 genes with insertion regions bias resolved by the sliding window method. Three of these proteins have unknown functions: VSP_RS0293 and VSP_RS40090, lack recognizable domains while VSP_RS39600 contains an essential domain of unknown function (DUF), DUF4340 (Data S6). Of the 34 pseudogenes, two are essential: VSP_RS43340 and VSP_RS43435, both being associated to the large ribosomal subunit, suggesting that they may be misannotated and represent functional genes rather than pseudogenes. The essential gene percentage of *V. spinosum* (10%) falls within the typical range for bacteria, such as the more remotely related *Escherichia coli* with 8%,^30^ but contrasts with the situation observed for *P. limnophila,* from the sister phylum *Planctomycetota*, with 17.8%, one of the highest observed to date.^17^

**Figure 1:**
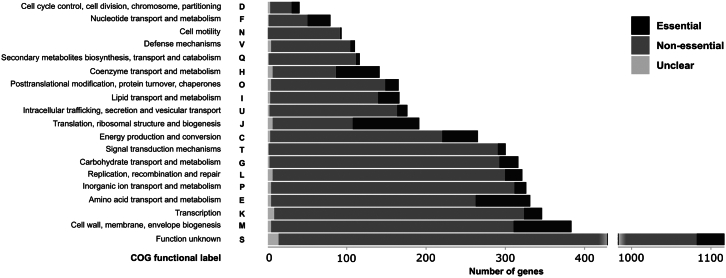
Distribution of COG orthology group functional labels by essentiality in *V. spinosum* The *x* axis shows the number of genes annotated with functional labels from EggNOG-mapper, as indicated on the *y* axis. For clarity, bars with higher values are truncated on the *x* axis.

*V. spinosum* proteome function and essentiality were analyzed using EggNOG-mapper to assign Clusters of Orthologous Groups (COG) labels. From the 6,166 proteins, 911 were not grouped in an orthology group, and 1,117 were annotated with the functional label “Function unknown” (S). A total of 3,002 proteins had a single functional label, excluding label S, while 404 had more than one and circa 700 orthology groups have no functional label. Most essential proteins were annotated with functional labels such as “Translation, ribosomal structure, and biogenesis” (J), “Cell membrane, wall, and envelope biogenesis” (M), and “Amino acid transport and metabolism” (E), followed by “Energy production and conversion” (C) (Figure 1). Of the 618 essential proteins, 100 are of unknown function, five of which also possess a DUF. Altogether, TraDIS provides crucial genome-wide essentiality data, making it a valuable first-line tool for any research topic.

Similar essentiality reports have been published for several strains, including *P. limnophila*^17^ and *E. coli* K12.^30^ To explore differences in essentiality, we carried out a comparison between *V. spinosum* and these organisms. Our analysis focuses on core essential genes, those likely to be indispensable under most growing conditions and typically involved in fundamental processes such as replication or division.^31^ Notably, essentiality screens in *P. limnophila*, *E. coli*, and *V. spinosum*, were all performed using rich media. These laboratory conditions represent less restrictive environments and thus, allow for a more accurate identification of core essential genes. In the *E. coli* K12 study by Goodall et al.,^30^ only CDS were considered, therefore we focused on the 4,258 CDS of *P. limnophila* (711 essential, 3,404 non-essential and 143 unclear) and the 6,166 CDS of *V. spinosum* (603 essential, 5,422 non-essential and 141 unclear). The genes identified through the essential domain correction method were excluded from this analysis, as this technique was not applied to *E. coli*. We used a bidirectional best-hit (bbh) method to identify orthologs between species, excluding proteins with paralogs due to possible complementation, as previously used.^17^

Between *V. spinosum* and *E. coli* 519 direct orthologs were identified (Data S7). Of these, 393 (75%) agree in their reported essentiality. Among the matching genes, 129 are essential, while 264 are non-essential. Most essential genes in both organisms are associated with “Translation, ribosomal structure and biogenesis” (J), “Coenzyme transport and metabolism” (H) as well as “Cell wall/membrane/envelope biogenesis” (M). These include genes that encode ribosomal proteins, RNA polymerase (*rpoBC*), and genes related to PG synthesis and cell division, such as *murADGIJ*, *mraY*, *ftsA* and *mreB*.

As for the comparison between *P. limnophila* and *V. spinosum*, 633 direct orthologs were found, 114 more than with *E. coli*, where 540 (85%) agreed in essentiality, reflecting the taxonomic closeness of both organisms (Data S8). Among these, 194 are essential, 345 are non-essential and one is unclear. Of the essential genes, the majority are classified into the functions of “Translation, ribosomal structure and biogenesis”, “Coenzyme transport and metabolism”, “Energy production and conversion”, and “Nucleotide transport and metabolism”. On the other hand, 93 genes do not match in reported essentiality between *P. limnophila* and *V. spinosum*. Among these, 47 essential genes in *P. limnophila* were identified that are not essential in *V. spinosum*. These genes are more diverse, and no single functional category predominates over the others. In addition, 23 were found to be essential in *V. spinosum* but not in *P. limnophila*, most of which are related to cell division and PG synthesis. *P. limnophila*, like most *Planctomycetota*, divides asymmetrically, via budding,^20^ while *V. spinosum* divides symmetrically via binary fission.^28^ This narrow range of genes not matching in essentiality emphasizes how division is one of the most distinctive features between these sister groups. The remaining 23 genes were unclear in at least one of the strains.

### Cell division in *V. spinosum*

**Figure 2:**
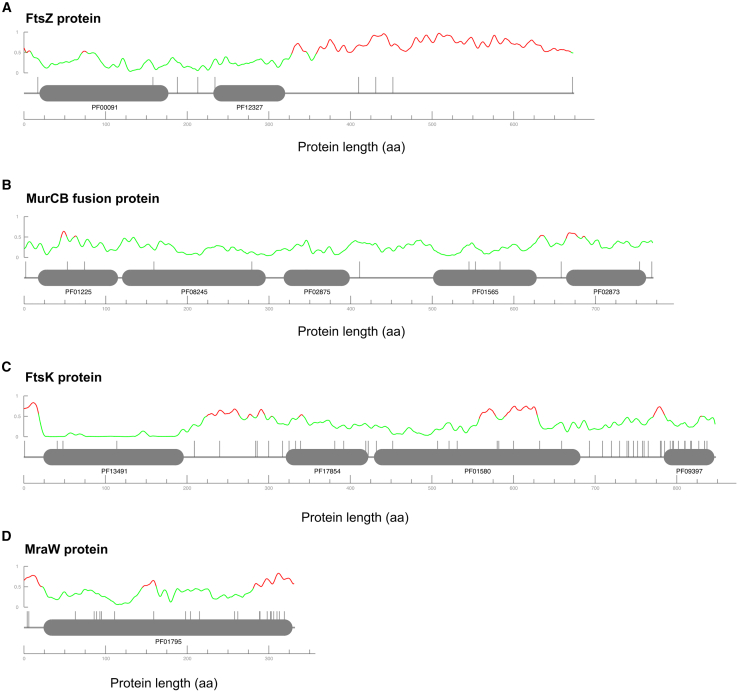
*V. spinosum* FtsZ, MurCB fusion domain FtsK and MraW organization The X axis represents the protein length in amino acids. The vertical lines represent transposon insertion sites. Pfam protein domains are shown in gray with the ID displayed below. The upper line represents the structural disorder predicted by IUpred3 on a scale from 0 to 1, where 0 is highly structured and 1 is unstructured. Regions predicted to have a structural disorder greater than 0.5 are shown in red, and those with less than 0.5 in green. (A) FtsZ protein (VSP_RS18465) with the Tubulin/FtsZ family domain, GTPase domain (PF00091), and the C-terminal FtsZ domain (PF12327). (B) MurCB protein (VSP_RS18435) with the Mur ligase domain (PF01225), Mur ligase intermediate domain (PF08245), and Mur ligase, glutamate ligase domain (PF02875), found in MurC, and the FAD-binding domain (PF01565) and UDP-N-acetyl-enolpyruvoylglucosamine reductase domain (PF02873), found in MurB. (C) FtsK protein (VSP_RS34595) with domains corresponding to PF01580 (FtsK/SpoIIIE family), PF17854 (FtsK gamma domain), PF13491 (FtsK DNA translocase) and PF09397 (SpoIIIE linker). (D) MraW protein (VSP_RS19480) containing the SAM-dependent methyltransferase domain (PF01795).

TraDIS analysis revealed that *murABCDEFGJ*, *ftsABIQWZ*, *mreBC*, *mraY*, *alr*, and *ddl* are essential in *V. spinosum*. In contrast, the homologs present in *P. limnophila* are not essential (Table 1). *V. spinosum* notable features include a C-terminus extension in FtsZ, an unusual characteristic that makes it one of the most divergent FtsZ homologs among bacteria.^32^ Despite being predicted as a disordered region, this extension is essential and tolerates very few insertions (Figure 2A). Another unique characteristic is the fusion of the *murC* and *murB* in a single gene, which was also found to be fully essential (Figure 2B). Interestingly, a functional *murC-ddl* fusion is also present in *C. trachomatis* serovar L2.^33^ These particularities of *V. spinosum* detected in *ftsZ* and *murCB* prevented them from being detected in the essentiality comparison, as they exceed the established query and subject coverage thresholds used for the identification of direct orthologs.

**Table 1:** Comparison of gene essentiality in the *dcw* cluster in *V. spinosum* and *P. limnophila*^17^

Gene	COG	*V. spinosum* NCBI locus tag	*V. spinosum* essentiality	*P. limnophila* NCBI locus tag	*P. limnophila* essentiality
*murA*	COG0766	VSP_RS11945	E	Plim_1195	NE
*murB*	COG0812	VSP_RS18435	E	Plim_0011	NE
*murC*	COG0773	VSP_RS18435	E	Plim_0012	NE
*murD*	COG0771	VSP_RS36300	E	Plim_1571	NE
*murE*	COG0769	VSP_RS36305	E	Plim_0681	NE
*murF*	COG0770	VSP_RS19460	E	Plim_0680	NE
*murG*	COG0707	VSP_RS19435	E	Plim_0205	NE
*murJ*	COG0728	VSP_RS28510	E	Plim_0195	NE
*ftsA*	COG0849	VSP_RS18470	E	–	–
*ftsB*	COG2919	VSP_RS17905	E	–	–
*ftsK*	COG1674	VSP_RS34595	U	Plim_2063	E
*ftsQ*	COG1589	VSP_RS18475	E	Plim_3909	NE
*ftsZ*	COG0206	VSP_RS18465	E	–	–
*mreB*	COG1077	VSP_RS17765	E	Plim_2620	NE
*mreC*	COG1792	VSP_RS17760	E	Plim_2621	NE
*alr*	COG0787	VSP_RS00105	E	–	–
*ddl*	COG1181	VSP_RS18480	E	Plim_0009	NE
*ftsW/rodA*	COG0772	VSP_RS36295/ VSP_RS37155	E/E	Plim_1127/Plim_1130	NE/NE
*ftsI/mrdA/pbp*	COG0768	VSP_RS19470/VSP_RS08025/VSP_RS17750	E/E/E	Plim_1789/-/Plim_4064	NE/-/NE
*mraW*	COG0275	VSP_RS19480	U	Plim_2101/Plim_4021	NE/NE
*mraY/wecA*	COG0472	VSP_RS19455/VSP_RS38955	E/E	Plim_1131/Plim_2437	NE/NE

E, essential; NE, non-essential; U, unclear; (−) Not present.

Within the *V. spinosum dcw* cluster, only *ftsK* and *mraW* were classified as unclear. In *E. coli*, FtsK is a multi-domain protein, with each domain performing distinct functions.^34^ The N-terminal domain and linker region are crucial for cell division, interacting with the early-acting FtsZ and the late-stage FtsQ, FtsL, as well as FtsW.^34^^,^^35^ The N-terminal domain is thought to be the only essential region,^36^^,^^37^ though some studies showed that its complete loss can be partially bypassed by the activity of the *ftsQAZ*.^38^ The C-terminal domain, on the other hand, functions as a DNA translocase and plays a key role in proper chromosome segregation during cell division.^39^^,^^40^^,^^41^ The size of the linker portion is highly variable, being large in *E. coli*^34^ but smaller in *V. spinosum.* In *V. spinosum*, a higher insertion density was observed in the C-terminal domain compared to the N-terminal domain (Figure 2C). This suggests that, similarly to *E. coli*, only specific domains of FtsK, particularly the N-terminal and possibly the linker regions, may be essential. Additionally, in *V. spinosum*, the *ftsQAZ* genes are clustered (Figure S5) and essential, suggesting that insertions in the N-terminal and linker regions of *ftsK* may be bypassed by proteins encoded from this cluster, similarly to other species.^38^ Interestingly, in its cousin species *P. limnophila*, *ftsK* is the only essential gene from the *dcw* cluster, further highlighting the diversification of cell division mechanisms within this superphylum.^42^ Unlike *E. coli* and *V. spinosum*, *P. limnophila* lacks both *ftsZ* and *ftsA*, making it impossible for FtsK function to be bypassed by FtsQAZ. FtsK in *P. limnophila* lacks the domain PF17854 (FtsK gamma domain) and, unlike in *V. spinosum* and *E. coli*, it does not contain non-essential domains. Its integrity is essential for its function.^17^ Recently, FtsK in *Chlamydia trachomatis* has been shown to be critical for its division,^43^ being an early recruited protein to the division site, a role typically performed by FtsZ. *C. trachomatis* and *P. limnophila* are known to divide asymmetrically in a budding-like mechanism,^44^ unlike *V. spinosum*, which divides symmetrically by binary fission. The link between the modification of function by FtsK and the absence of FtsZ in the asymmetrical division mechanisms observed in both *Planctomycetota* and *Chlamydiota*, is still unclear.

Similarly to *ftsK*, *mraW* essentiality was impossible to determine through the applied method due to the frequency and location of transposon insertions (Figure 2D). MraW is an RNA methyltransferase that specifically targets the 16S ribosomal RNA.^45^ While its primary role is largely linked to translation fidelity, DNA methylation,^46^ and regulation of MraZ,^45^ null mutants in *E. coli*, remain viable and display minimal phenotypic effects.^46^

To evaluate gene essentiality, a *V. spinosum* mutant strain (DV114) with the *mraW* gene knockout was constructed. Prior to the generation of DV114, protocols for targeted gene deletion were first developed using non-essential genes, as targeted mutagenesis had not previously been established for *Verrucomicrobiota*; described in the method details section “Targeted mutagenesis in *V. spinosum*” and Figure S6. The viability of DV114 confirmed that *mraW* is non-essential under the conditions used. Interestingly, in *V. spinosum*, *mraW* is located in a large essential cell division and peptidoglycan cluster (Figure S5).

### Peptidoglycan composition in *V. spinosum*

**Figure 3:**
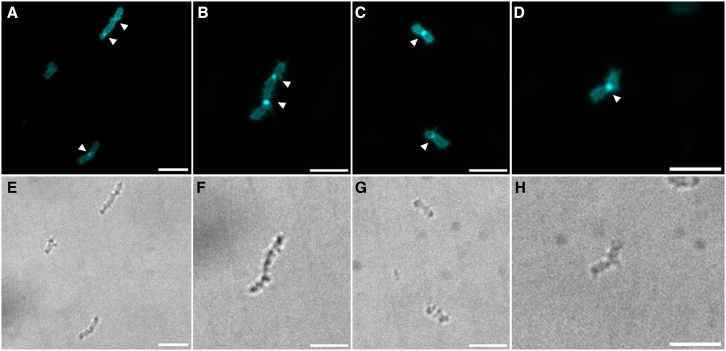
HADA staining of *V. spinosum* (A–D) Fluorescence confocal microscopy images. (E–H) Corresponding brightfield images. Strains were grown exponentially and stained with HADA. Septal incorporation is indicated by an arrow. Scale bars represent 5 μm.

Controversy has surrounded PG composition and presence in PVC members.^13^ Fueling the controversy, most PG synthesis genes are not essential in the cousin lineage *P. limnophila*.^17^ We reveal that those genes are essential in *V. spinosum*, potentially suggesting a classical PG synthesis and composition. To evaluate PG synthesis, exponentially grown *V. spinosum* cells were labeled using the fluorescent D-amino acid 7-hydroxycoumarincarbonylamino-*d*-alanine (HADA). This approach allowed to visualize sites of active PG incorporation in real-time, providing insights into the spatial and temporal dynamics of cell wall biosynthesis. PG synthesis occurred predominantly at the septal plane (Figure 3), similarly to results achieved in Kuru et al.^47^ Elongated cells were also seen actively dividing with multiple septal synthesis co-occurring.

**Figure 4:**
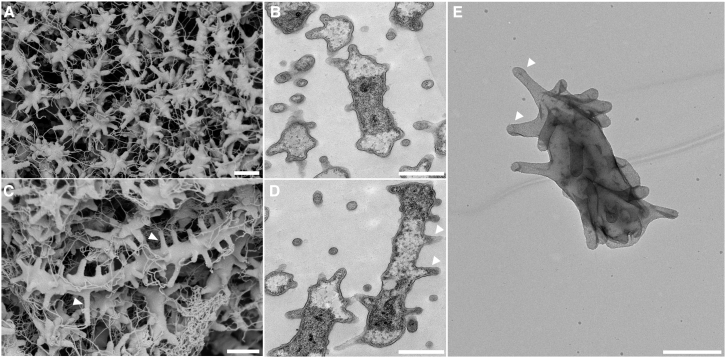
Electron microscopy of *V. spinosum* (A and C) Scanning electron microscopy of *V. spinosum.* (B and D) Transmission electron micrographs of *V. spinosum.* Images were taken during exponential (A and B) and stationary growth (C and D). Prosthecae are indicated by arrows. (E) Negative staining of *V. spinosum* sacculi. Scale bars represent 1 μm.

To evaluate the presence of a saccular PG, *V. spinosum* PG was extracted using standard SDS-boiling protocols and visualized by negative staining under electron microscopy. Additionally, sacculi images were compared to cell imaging obtained by scanning (SEM) and transmission (TEM) electron microscopy. *V. spinosum* exhibits morphological pleomorphism, were bacteria range from short to elongated rod-like cells (Figure 4). Elongated cells were observed throughout the growth curve, though a higher prevalence was observed during the late exponential and stationary phases. Characteristic prosthecae of various lengths were visible throughout the cell cycle. Additionally, multiple fimbriae were observed extending from the tips of each prosthecae. *V. spinosum* cells possesses a saccular PG that retains the unique morphology of the cell prostheca (Figures 4; S7).

Despite partial characterisation,^24^ complete composition of the *V. spinosum* PG was lacking. Here, the analysis of purified PG from *V. spinosum* showed a canonical composition containing as major muropeptides the disaccharides tripeptide (peak 4), tetrapeptide containing glycine (peak 5) and tetrapeptide containing alanine (peak 6), and the bis-disaccharides tritetrapeptide (peak 14) and tetratetrapeptide (peak 15) (Figure S8). Most of the peaks analyzed by MS and MS/MS contain non-acetylated glucosamine (GlcNH_2_), with exception of the peak 2 (disaccharide GlcNAc-MurNAc, GM) and peak 8 (disaccharide tetrapeptide, GM-AEmA).

Non-acetylated glucosamine residues in PG have been identified in a few organisms, including notable pathogens such as *Bacillus anthracis*, *Bacillus cereus*,^48^ *Listeria monocytogenes*^49^ and *Streptococcus pneumoniae*.^50^ In some of these, this modification has been associated with lysozyme resistance,^48^ as well as a mechanism for evading recognition by the immune system.^49^ Interestingly, *A. muciniphila*, a symbiont close relative of *V. spinosum*, has also been shown to possess PG containing non-acetylated glucosamine residues.^23^ Although *V. spinosum* has been implicated as a potential pathogen in invertebrates such as *Drosophila melanogaster* and *Caenorhabditis elegans*, there is no evidence suggesting pathogenicity in vertebrate organisms.^51^ Moreover, *V. spinosum* is commonly found in environments like lakes^28^ and rhizospheres.^52^ While research has primarily focused on the role of non-acetylated PG in pathogenic bacteria, possibly introducing a bias, its functions in environmental bacteria and beneficial symbionts remain largely unexplored. Given that there are only two reports on the PG composition within *Verrucomicrobiota*,^23^^,^^24^ in addition to the present study, non-acetylated glucosamine PG might represent a characteristic feature of this phylum. Nonetheless, many other organisms are also capable of deacetylate glucosamine through deacetylase activity, an activity found in almost all bacteria, according to the orthologs distribution (COG0726), reviewed in Vollmer.^53^

Altogether, the data presented here, including *dcw* genes essentiality, HADA incorporation, sacculi structure, and PG composition confirm that *V. spinosum*, probably like most other Verrucomicrobiota, divides by binary fission in a canonical manner, using “classical” PG and FtsZ.

### Conservation and essentiality of dcw genes in the PVC superphylum

**Figure 5:**
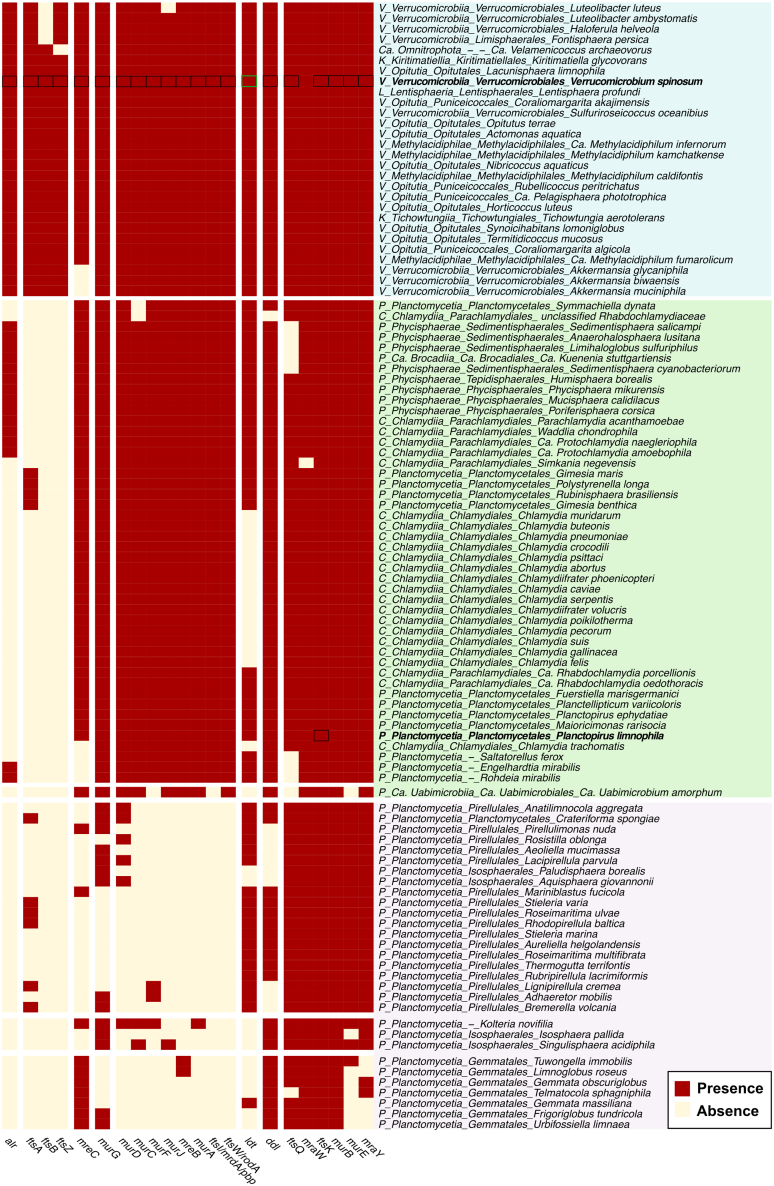
Conservation of the *dcw* cluster in the PVC superphylum Heatmap of orthologous groups from a selection of *dcw* cluster genes for the reference proteomes of PVC in NCBI. Each row represents one of the selected proteomes. Each column represents an orthology group of the selected dcw cluster genes. The presence of an orthology group in a specific proteome is represented in red, while its absence is shown in light yellow. The main clusters are shaded in purple, blue and green. Essential genes are surrounded by a black square. The green box represents the impossibility to determine essentiality due to numerous paralogs *P*- *Planctomycetota*, *V*- *Verrucomicrobiota*, *C*- *Chlamydiota*, *K*- *Kiritimatiellota*, *L*- *Lentisphaerota*, and *Ca.*- *Candidatus*.

Phylogenetic profiling of ortholog groups within the *dcw* cluster in PVC superphylum was contrasted with the essentiality data given by *V. spinosum* and *P. limnophila*. These analyses revealed a diverse pattern of conservation within the cluster. Those genes are highly conserved in the phyla *Verrucomicrobiota*, *Candidatus* Omnitrophota, *Kiritimatiellota*, and *Lentisphaerota* (cluster in blue, Figure 5). Another major group comprises the phylum *Chlamydiota*, and the *class Phycisphaerae* and the order *Planctomycetales* (class *Planctomycetia*) from the *Planctomycetota* phylum (cluster in green), where gene conservation is nearly complete, except for the loss of *ftsZ*, *ftsB*, *ftsA*, and *alr,* with few exceptions for the latter two genes. Lastly, there is a set within the class *Planctomycetia* formed by the orders *Pirellulales*, *Isosphaerales*, and *Gemmatales* (cluster in purple) that shares the same absences as the previous one, but they have also experienced losses of genes related to PG synthesis. Specifically, they have lost most genes associated with the cytoplasmic synthesis of lipid II precursors, *murA*, *murC*, *murD*, *murF*, *and murG,* as well as those involved in the transport of lipid II to the periplasm, *murJ* and *ftsW*. The only genes from the *dcw* cluster conserved throughout the PVC superphylum are *murB*, *mraW*, and *ftsK*.

In *V. spinosum*, where these genes are highly conserved (cluster in blue) and division takes place by binary fission, most of the genes including *murABCDEFGJ*, *ftsABIQWZ*, *mreBC*, *mraY*, *alr*, and *ddl*, are essential. By contrast, in *P. limnophila*, *murABCDEFGJ*, *mreBC*, *ftsIKQW*, *mraWY*, *ddl*, and *ldt*, despite being conserved in the order *Planctomycetales* (cluster in green), are not essential, with the exception of *ftsK* (Table 1). This discrepancy could be attributed to the existence of different division modes within the cluster, possibly showcasing intermediate stages between the other clusters. Additionally, the cluster in green, contains organisms in which the remaining *dcw* genes are essential like *Chlamydiota*,^21^^,^^43^ and others, like *P. limnophila*, in which those genes are not essential. Probably, the lack of essentiality in *Planctomycetia* is due to the emergence of an alternative cell division mechanism not based on *ftsZ* and not dependent on PG synthesis genes, which concluded in the loss of these genes in the orders *Pirellulales*, *Isosphaerales*, and *Gemmatales*. Hence, the conservation patterns of these proteins and the essentiality of the genes encoding them can only be fully understood considering the diversity of their division mechanisms.

Considering the presence of most *dcw* genes in phyla such as *Verrucomicrobiota*, *Ca.* Omnitrophota, *Kiritimatiellota*, and *Lentisphaerota*, along with the coexistence of binary fission, suggests that the LPCA possessed a division mechanism similar to that of other Gracilicutes. From this ancestral mechanism, new FtsZ-independent division systems have subsequently evolved. This divergence is likely related to the fragmentation of the *dcw* cluster.^16^

### Limitations of the study

This study is the first to characterize gene essentiality in *V. spinosum*. While the mutant library provides valuable insight into gene essentiality, it does not account for multiple simultaneous mutations or compensatory mechanisms. Findings are also limited to optimal lab conditions, and gene essentiality may vary under different environments. Despite this, core genes remain invariable under most conditions. The current analysis, conducted under ideal growth conditions with minimal constraints, offers a useful reference for future condition-specific studies. Lastly, we developed the first tools for targeted mutagenesis in *Verrucomicrobiota*, laying the groundwork for genetic manipulation in the phylum. However, more sophisticated methods, such as scarless deletions, replicative plasmids, and inducible promoters, are yet to be developed.

## Resource availability

### Lead contact

Requests for further information and resources should be directed to and will be fulfilled by the lead contact, Elena Rivas-Marin (elenarivasmarin@gmail.com).

### Materials availability

All unique/stable reagents generated in this study are available from the lead contact with a completed materials transfer agreement.

### Data and code availability

• The raw sequencing data from the TraDIS library and the WGS of DV085 generated in this study are available through NCBI Bioproject under accession number PRJNA1267754.
• The custom scripts used for analyses in this study are available in the GitHub repository: https://github.com/dmoypal/TraDIS_in_V.spinosum.
• All other data generated in this study are provided in the supplemental information and Data files. Source data are provided in this paper.

## Acknowledgments

Thanks to Cristina Vaquero Aguilar (CITIUS Universidad de Sevilla Microscopy Facility) for electronic microscopy services, Laura Tomás (CABD Proteomics and Biochemistry Unit) for proteins purification services, and Katherina García (CABD Advanced Light Microscopy and Image Analysis Facility) for light microscopy services. Ildefonso Cases (CABD computational unit) for bioinformatic services. V.H. is funded by the Portuguese national funds through FCT-Fundação para a Ciência e a Tecnologia, Portugal (2022.11400.BD); D.M.-P. by Junta de Andalucía Predoctoral Grant (Predoc_00913); M.P. is an Atracción de Talento M1 Fellow from Comunidad de Madrid (Spain) [2020-T1/BMD-19970] who receives support [PID2022-140818OA-I00] from the Spanish Ministry of Science, Innovation and Universities (MICIU/AEI/https://doi.org/10.13039/501100011033 and ERDF/EU); D.P.D. and E.R.-M. by Mineco (Grant No. PID2020-119733GBI00) and the “Moore-Simons Project on the Origin of the Eukaryotic Cell” (Grant No. 9733/https://doi.org/10.37807/GBMF9733).

## Author contributions

V.H. and E.R.-M. performed the molecular biology, culturing optimizations, constructed the insertion and deletion mutants, the transposon mutant library, and physiological characterizations. D.M.-P. and D.P.D. performed *in silico* analysis. M.T.A.-P. and M.P. determined peptidoglycan composition. E.R.-M. and D.P.D. designed the study. V.H., D.M.-P., E.R.-M., D.P.D., and M.P. interpreted the results and discussed the analyses. V.H., D.M.-P., E.R.-M., D.P.D., and M.P. wrote the manuscript. All authors read and approved the final manuscript.

## Declaration of interests

The authors declare no competing interests.

## STAR★Methods

### Key resources table

**Table:** 

REAGENT or RESOURCE	SOURCE	IDENTIFIER
**Bacterial and virus strains**		

*Escherichia coli* DH5α Genotype: F^-^ ϕ80*lacZ*ΔM15 Δ(*lacZYA-argF)U169 recA1 endA1hsdR17*(r_K_^-^m_K_^*-*^) *supE44 thi-1 gyrA relA1*	Laboratory collection	Hanahan^54^
*E. coli* ER2566 Genotype:*fhuA2 lacZ::T7 gene1 [lon] ompT gal sulA11 R(mcr-73::miniTn10--*Tet^S^*)2 [dcm] R(zgb-210::Tn10--*Tet^S^*) endA1 Δ(mcrC-mrr)114::IS10*	New England Biolab	Catalog #: C2566H
*Verrucomicrobium spinosum*	DSMZ	DSM 4136
*V. spinosum* DV086 Genotype:*VSP_RS42430*::pDV134	This work	N/A
*V. spinosum* DV025 Genotype: Δ*VSP_RS24925.* Km^R^	This work	N/A
*V. spinosum* DV085 Genotype:*VSP_RS24925*’::Tn*5*. Km^R^	This work	N/A
*V. spinosum* DV114 Genotype: Δ*mraW.* Km^R^	This work	N/A

**Chemicals, peptides, and recombinant proteins**		

HADA	Bio-Techne (TOCRIS)	Cat. No. 6647
Tn*5* transposase	This work	N/A
SDS ≥99.0%	Sigma-Aldrich	REF: 71726

**Critical commercial assays**		

NucleoSpin Plasmid miniprep kit	Macherey-Nagel	REF: 740588.250
DIG DNA Labeling Kit	Roche	REF: 11175033910
illustra™ GFX™ PCR DNA and gel band purification kit	Cytiva	REF: 28-9034-71
Wizard® Genomic DNA Purification Kit	Promega	REF: A1120
Anti-Digoxigenin-AP, Fab fragments	Roche	REF: 11093274910
CSPD	Roche	REF: 11655884001

**Deposited data**		

Sequencing data from the TraDIS	This work	Database: PRJNA1267754 (NCBI BioProject)
WGS DV085	This work	Database: PRJNA1267754 (NCBI BioProject)
Custom scripts used for analyses	This work	https://github.com/dmoypal/TraDIS_in_V.spinosum
Optical density from growth curves (see Data S1. Excel file containing OD600 values used for graphical representation of Supplementary Figure 1A–1C, Data S2. Excel file containing OD600 values used for graphical representation of Supplementary Figure 1D, Data S3. Excel file containing OD600 values used for graphical representation of Supplementary Figure 1E)	This work	N/A
Muropeptides detection (see Data S10)	This work	N/A

**Oligonucleotides**		

Oligonucleotides (See Table S3)	Merck	N/A

**Recombinant DNA**		

Plasmids (See Table S2)	This work	N/A

**Software and algorithms**		

FIJI (v1.54f)	Schindelin et al.^55^	https://github.com/fiji/fiji
SlideBook (v6)	https://www.intelligent-imaging.com/slidebook	N/A
Trimmomatic (v0.32)	Bolger et al.^56^	https://github.com/timflutre/trimmomatic
bowtie2 (v2.5.1)	Langmead and Salzberg^57^	https://github.com/BenLangmead/bowtie2
SAMtools (v1.17)	Li et al.^58^	https://github.com/samtools/samtools
picard-2 (v3.0.0)	https://broadinstitute.github.io/picard/	N/A
IGV genome browser (v2.16.1)	Robinson et al.^59^	https://igv.org
MASS (v7.3-60)	https://cran.r-project.org/web/packages/MASS/index.html	N/A
Fitdistrplus (v1.1-11)	Delignette-Muller and Dutang^60^	https://cran.r-project.org/web/packages/fitdistrplus/index.html
R (v3.10.12)	https://www.r-project.org	N/A
Python (v3.10.12)	https://www.python.org	N/A
EggNOG-mapper (v2.1.12)	Cantalapiedra et al.^61^ Huerta-Cepas et al.^62^	https://github.com/eggnogdb/eggnog-mapper
protein BLAST (2.12.0+)	Camacho et al.^63^	https://blast.ncbi.nlm.nih.gov/Blast.cgi
Pheatmap (v1.0.12)	Kolde, Pheatmap: Pretty Heatmaps. R package version 1.0.12 (2019)	https://cran.r-project.org/web/packages/pheatmap/index.html
Prism (v 8.0.2)	GraphPad	N/A

### Experimental model and study participant details

#### Escherichia coli

*E. coli* DH5α was used for plasmid propagation detailed in Table S2. *E. coli* ER2566 bearing the plasmid pTXB1-Tn*5* was used for transposase production. *E. coli* strains were grown in Lysogeny broth medium (LB) at 37°C and 180 rpm with appropriate antibiotics and inducers when required.

#### Verrucomicrobium spinosum

*V. spinosum* DSM 4136 wild type strain was obtained from DMSZ. This wild type strain was used as background for the Tn*5*-mutant library construction, as well as to construct DV086, DV025 and DV114 strains. An individual randomly-selected mutant from the Tn*5*-mutant library was picked and subject to whole genome sequencing to verify single insertion event of the Tn*5* transposon, this strain was named DV085 and WGS revealed a single insertion in the VSP_RS24925 gene. DV086 bears an insertion of the plasmid pDV134 in the end portion of VSP_RS42430 gene. DV025 and DV114 are deletions strains for VSP_RS24925 and *mraW* genes respectively. More details for the plasmids and oligonucleotides used for the development of targeted mutants can be found at Tables S2 and S3 in supplemental information. *V. spinosum* culturing medium was improved in the current study, optimizations are detailed in the methods details section. The composition of final medium is as follows (w/v): 0.025% yeast extract, 0.025% peptone, 0.005% ammonium chloride, 10 mM HEPES (pH 8), 0.1% NAG, 0.25% glucose, 25% (v/v) artificial sea water from DSMZ 607 medium, 1% (v/v) vitamin solution from DSMZ 603 medium and 2% (v/v) Hutner’s basal salt solution from DSMZ 590 medium. 1% bacto-agar was added for the solid medium. Cultures were grown aerobically in a shaker (180 rpm) at 30°C with appropriate antibiotics when required.

### Method details

#### *V. spinosum* medium optimisation

For optimisation of *V. spinosum* growth conditions, it was evaluated the addition of N-acetyl-glucosamine (NAG) and ammonium chloride, and the increment of glucose concentration to the described DSMZ medium 607 (M13) [http://www.dsmz.de]. Growth curves were performed in the presence of different concentrations of glucose, NAG and ammonium chloride in a microplate where the optical density was measure at 600 nm every 1.5 h in a Tecan Spark® multimode microplate reader. Microplates were incubated aerobically at 180 rpm and 30°C. Every condition was performed in replicates and results are summarized in Figure S1. The composition of final medium is as follows (w/v): 0.025% yeast extract, 0.025% peptone, 0.005% ammonium chloride, 10 mM HEPES (pH 8), 0.1% NAG, 0.25% glucose, 25% (v/v) artificial sea water from DSMZ 607 medium, 1% (v/v) vitamin solution from DSMZ 603 medium and 2% (v/v) Hutner’s basal salt solution from DSMZ 590 medium. 1% bacto-agar was added for the solid medium. Cultures were grown aerobically in a shaker (180 rpm) at 30°C.

### Antibiotic susceptibility test

Antimicrobial resistance profile was performed by plating serial dilutions of a 3-day saturated culture in solid media containing cycloheximide 50 μg.mL^−1^, to avoid fungal contaminations, plus the following antibiotics, and respective concentrations: ampicillin (25, 50, 100, 200 μg.mL^−1^), gentamicin (5, 10, 20 μg.mL^−1^), streptomycin (25, 50, 100 μg.mL^−1^), kanamycin (5, 10, 25, 50 μg.mL^−1^), tetracycline (2.5, 5, 10 μg.mL^−1^) and chloramphenicol (5, 10, 20, 30 μg.mL^−1^). Plates were incubated for 7 days. Results were compiled in Figure S2. Chloramphenicol 5 μg.mL^−1^ and cycloheximide 50 μg.mL^−1^ were used for routinely cultivation.

### Targeted mutagenesis in *V. spinosum*

Currently there is no mention of targeted mutagenesis in the phylum *Verrucomicrobiota*. Therefore, we develop and applied a protocol for both insertion and deletion in *V. spinosum*. The plasmids and oligonucleotides used in this work are summarised in Tables S2 and S3, respectively.

To generate the insertion mutant DV086 via one-event homologous recombination, the plasmid pDV134 was introduced via electroporation into competent *V. spinosum* cells. In order to support this homologous recombination, a fragment of 907 bp of *V. spinosum* endonuclease gene (*VSP_RS42430*) was amplified using genomic DNA as a template and was cloned into the pMPO1012 as a HindIII fragment to generate pDV134. For genetic transformation, fresh electrocompetent cells were prepared from 400 mL of a culture at an OD_600_ of 0.3-0.4 in modified M13. The cells were washed twice with 100 and 50 mL of ice-cold double distilled sterile water and once with 2 mL of ice-cold 10% glycerol. Then, the pellet was resuspended in 400-500 μL of ice-cold 10% glycerol, and aliquots of 100 μL were made. Electrocompetent cells were dispensed into 0.1-mm gapped electroporation cuvettes along with around 1 μg of purified plasmid and electroporated with EC3 pulse (3.0 kV). All electroporations were performed with a BioRad Micropulser. Electroporated cells were immediately recovered in 1 ml of cold modified M13 and incubated at 30°C for 2 h with shaking. The cells were then plated onto modified M13 plates supplemented with kanamycin 50 μg.mL^−1^ and incubated at 30°C until colony formation after 5 to 7 days. Colonies were segregated onto fresh selection plates and genotyped by southern blotting analysis and PCR. Transformants were selected with kanamycin, while no colonies appeared in negative controls. Integration frequency was ∼10^-6^ cfu to 10^-7^ cfu/μg per recipient. The insertion mutant was confirmed by southern blotting (Figure S6A).

The generation of deletion mutants was also assayed by targeting the gene *VSP_RS24925* (annotated as a beta-ketoacyl synthase N-terminal-like domain-containing protein) via double homologous recombination (DV025). In more detail, the plasmid pDV201 containing 968 and 960 bp of upstream and downstream fragments, respectively, of the *V. spinosum* beta-ketoacyl synthase gene (*VSP_RS24925*) were amplified by PCR from genomic DNA using the primer pairs listed in Table S3. The upstream and downstream fragments were digested with HindIII/BamHI and EcoRI/BamHI respectively and cloned into pEX18Tc by two-way ligation. Finally, the kanamycin resistance gene was amplified from the pUTminiTn5km and subsequently cloned as a BamHI fragment between the two flanking regions. Electroporation of the plasmid into competent *V. spinosum* cells was performed similarly as aforementioned to produce the deletion mutant. Transformants were selected with kanamycin, no colonies appeared in negative controls. Integration frequency was ∼10^-7^ cfu/μg per recipient, with double recombination events occurring directly. The *VSP_RS24925* deletion was confirmed by PCR (Figure S6B). For the DV114 *mraW* knockout mutant the plasmid pDV214 was transformed by electroporation through the same protocol applied for the DV025 deletion mutant. This plasmid possessed an upstream (858 bp, EcoRI/BamHI) and downstream (815 bp, HindIII/BamHI) region of the *V. spinosum mraW* gene (*VSP_RS19480*) and a kanamycin resistance gene (BamHI) cloned into pEX18Tc in a three-way ligation. Transformants were selected with kanamycin, no colonies appeared in negative controls.

Attempts to create scarless mutants with a plasmid containing joined flanking regions were, however, unsuccessful. These advancements represent the first targeted mutation in the phylum, expand the research possibilities and establish *V. spinosum* as the model organism for the *Verrucomicrobiota*. Nonetheless, the development of replicative plasmids and tightly regulated promotors are still areas to be addressed in future work.

#### Southern blotting and PCR mutant confirmation

Genomic DNA from DV086, DV025 and DV114 strains were extracted using Wizard Genomic DNA Purification Kit (Promega). DV086 mutant was confirmed by southern blotting using 2 μg of genomic DNA. The digested DNA was resolved by agarose gel electrophoresis and the DNA transfer was performed as described previously.^64^ PCR amplicon of the endonuclease end portion area was used as probe and amplified with the same primers used for genomic amplification for DNA cloning (Table S3). Probes were labelled according to the manufacturer’s instructions (DIG DNA Labeling Kit, Roche). DIG-labelled probes were detected with anti-Digoxigenin-AP, Fab fragments (Roche) and CSPD (Roche). Visualization was performed using ChemiDoc MP (BioRad). DV025 mutant was confirmed by PCR using two distinct primer pairs and subsequent restriction enzyme digestion, graphical representation in Figure S6B. Specifically, for the first PCR, the primers LFR_Whi_fwd (internal primer) and Out DV025_rv (external primer) were used to amplify a region of 3032 bp in the wild type (WT) and 3244 bp in the DV025 mutant. In the second PCR, the primers RFR_Whi_rv (internal primer) and Out DV025_fw (external primer) amplified a region of 3029 bp in the WT and 3241 bp in the mutant. Both PCR reactions occurred with annealing temperature of 64°C, extension time of 1 min 50 sec and DMSO 5%. Given the similar sizes of the amplification products, the PCR products from both reactions were subjected to digestion with the restriction enzyme PstI. This digestion yielded two fragments in the WT sample (2445 bp and 587 bp for first PCR and 2481 bp and 548 bp for the second) and three fragments in the DV025 mutant (1603 bp, 1054 bp and 587 bp for first PCR and 1639 bp, 1054 bp and 548 bp for the second). DV114 mutant was confirmed by PCR using the external primers Out DV114 fw and Out DV114 rv to amplify a region of 3037 bp in the mutant and 2706 bp in the WT. PCR reactions occurred with annealing temperature of 61C, extension time of 1 min 42 sec and DMSO 5%.

#### Transposase purification and assembly

The transposase was purified by the Proteomic facility at the CABD (Seville, Spain) according to Picelli et al.^65^ with modifications in detailed described in Rivas-Marín et al.^17^ Transposome assembly was performed following the protocol described by Goryshin et al.,^66^ with some modifications. Transposomes were formed by incubating kanamycin transposon DNA (up to 50 μg.mL^–1^) with 15 μg.mL^–1^ Tn*5* transposase for 1h at 37°C in a 40 μL reaction volume in transposon buffer (27.5 mM Tris-HCl, pH 7.5, 50 mM NaCl, 0.075 mM EDTA, 0.5 mM DTT, 0.05% Triton X-100, and 50% glycerol). Transposomes were stored at −20°C until used. Kanamycin gene was amplified using primers Km IS fwd and Km IS rv (Table S3).

#### Transposon library construction

*V. spinosum* WT was used for the construction of a transposon library. Fresh electrocompetent cells were prepared as described above. Aliquots of 100 μL of competent cells were dispensed into 0.2-mm gapped electroporation cuvettes along with 1 μL of transposome and electroporated using EC3 pulse (1.5 kV). Electroporated cells were immediately recovered in 1 mL of cold-modified M13 medium and incubated at 30°C for 2 h with shaking. Transposon mutants were selected by growth onto modified M13 supplemented (100 mm plates) with cycloheximide 50 μg.mL^-1^ and kanamycin 50 μg.mL^-1^ at 30°C during for 10 days. Plates were swabbed and approximately 1.25 million colonies were pooled and stored at -80°C. Clones were not replica plated prior to harvest, thus, clones with limited growth may have also been included. To verify Tn*5* single insertion and its location, genomic DNA was extracted from the transposon mutant DV085 and subject to whole-genome of sequencing by Illumina PE150 by Novogene (UK) Company Limited (United Kingdom). Genomic DNA from DV085 was extracted using the Wizard Genomic DNA Purification Kit (Promega). The concentration and quality of the genomic DNA were checked spectrophotometrically and on agarose gel electrophoresis, respectively.

#### Library preparation and sequencing

DNA was collected from 19 independent genomic extractions of the transposon library to generate TraDIS data. The extractions were performed by using the Wizard Genomic DNA Purification Kit (Promega). The concentration of the genomic DNA was checked using Picogreen method (Qubit) and the quality by agarose gel electrophoresis. Eight libraries were sent for preparation and sequencing by Illumina MiSeq to Fasteris (Switzerland). For data quality control, Spikes-PhiX and Q30 were used. Trimmomatic was also used to filter reads based on quality.

#### Sequencing data mapping

Fasteris post-processed raw data, removing adapters from sequencing using Trimmomatic (v0.32)^56^ command “adapterFile:2:30:10:1:true SLIDINGWINDOW:4:5” with the adapterFile containing the following adapter sequences: “ATCTCGTATGCCGTCTTCTG”, “TGGAATTCTCGGGTGCCAAG”and “CTGTAGGCACCATCAAT”. Reads with the transposon were positive selected based on the primer sequence “GTTCGAAATGAGATGTGTATAAGAG” and transposon tail “ACAG”, and primer and tail were removed from the reads, retaining only reads longer than 20 bp using Trimmomatic (v.032). The resulting reads were mapped to the genome (RefSeq accession NZ_ABIZ01000001.1) with bowtie2 (v2.5.1).^57^ The subsequent steps of conversion from SAM (sequence alignment/map) files to BAM (binary version of SAM) files, and the requisite sorting and indexing, were done using SAMtools (v1.17).^58^ Duplicated reads were removed with picard-2 (v3.0.0) (https://broadinstitute.github.io/picard/). Data were inspected manually using the IGV genome browser (v2.16.1).^59^

#### Essential gene prediction

We followed the method proposed by Goodall et al.,^30^ with the modification of Rivas-Marin et al.^17^ Insertion rate was calculated as the total number of insertions per gene divided by the total gene size. As in the reference, the distribution presented two modes. Each one was adjusted to a different model, the first section was adjusted to the exponential distribution and the second to the gamma distribution using packages MASS (v7.3-60) and fitdistrplus (v1.1-11) in R (v3.10.12). Unlike the original method, the distribution was separated into two sections to minimise overlapping in the transition between modes. The insertion index cut-off values between these sections were manually established in 0.006 and 0.03, based on the observation of data. The likelihood that a gene belonged to each of these two distributions was calculated. The ratio of both likelihoods was used to calculate a log-likelihood score. Genes were assigned as essential by TraDIS if the log of the likelihood ratio is higher than log_2_ (12), as non-essential if this is less than log_2_ (-12), and unclear if this is between these values.

A Python (v3.10.12) script was used to interrogate the TraDIS unclear and non-essential genes, published in Rivas-Marin et al.^17^ A window of 300 bp started the insertion count at the beginning of the gene and slid every 150 bp. A window was labelled as essential when no inserts were found inside the window.

#### Distribution of *V. spinosum* genes by functional class

The annotation of COG functional label from *V. spinosum* was obtained using EggNOG-mapper (v2.1.12).^61^^,^^62^ This annotation was plotted in different bar charts using the packages ggplot2 (v3.4.2) in R (v3.10.12). The COG functional labels from genes with more than one label were separated and taken into account as different to count the number of total labels.

#### Comparative essentiality of *V. spinosum*, *P. limnophila* and *E. coli* direct orthologs

A reciprocal protein BLAST (BLASTP version 2.12.0+)^63^ was carried out one by one between *V. spinosum* (accession no. NZ_ABIZ01000001.1), *P. limnophila* (accession no. CP001744.1 and CP001745.1) and *E. coli* K-12 BW25113 (accession no. CP009273.1) proteomes. The results were filtered using a cut-off E-value of 1 × 10^-5^ and a query and subject coverage of at least 70%. We then applied additional filter criteria to remove proteins with paralogs. The ortholog proteins found were classified based on the comparative essentiality reported.

#### Phyletic distribution of *dcw* cluster PVC superphylum

The proteomes of PVC superphylum was retrieved from NCBI Genome, selecting only reference complete genomes (downloaded May 2025) (Data S9). These proteomes were annotated using EggNOG-mapper (v2.1.12)^61^^,^^62^ using the default parameters. For each of the ortholog groups belonging to the *dcw* cluster, the presence of each one in every proteome was detected. For those groups that appeared more than once in the same organism, only one occurrence was considered to denote their absence or presence. With the presence/absence data of orthologous groups in the different organism, a heatmap was made using package Pheatmap (v1.0.12) (Kolde, Pheatmap: Pretty Heatmaps. R package version 1.0.12 (2019)). A hierarchical clustering was made with the complete linkage method and Euclidean distance by this library. The number of clusters was designated manually based on tree observations.

#### Peptidoglycan isolation and analysis

For PG extraction, a 3-day pre-inoculum of *V. spinosum* was upscale to 50 mL and then to 2.4 L in modified M13 medium with cycloheximide 50 μg.mL^−1^ at 30°C and 180 rpm. Cells were cooled on ice for 15 min and collected at 8.000 *xg* for 30 min at an OD_600_ of 1. The pellet was resuspended in 36 mL of cold double distilled water and lysed by adding the resuspended pellet, dropwise, to 36 mL of boiling 8% SDS ≥99.0% (Sigma-Aldrich 71726) within 40 min, under vigorous stirring. A second and a third boiled took place for an additional 30 min each, sample was cooled to room temperature and added 0.02% of sodium azide. Samples were pelleted by centrifugation at 100.000 *xg* and washed with bidistilled water several times to remove all the SDS. SDS presence was detected following Hayashi test.^67^ Pellets were resuspended in 2 mL of 10 mM Tris/HCl, 10 mM NaCl, pH 7.0, and incubated with 400 μg of α-amylase at 37°C, 1.000 rpm, for 4 h. Sample was incubated for 2 h with 400 μg of pronase E at 37°C, 1.000 rpm, mixed with 4% SDS HPLC-grade (1:1 v/v) and boiled for 15 min at 100°C. After cooling down at room temperature, sample was washed with bidistilled water until become SDS free. Isolated PG was resuspended in a final volume of 2 mL of 0.02% NaN_3_ and store at 4°C.

For LC-MS/MS samples a previously published protocol was followed with minor modifications.^23^ Briefly, 200 μL of PG sample was incubated (overnight, 37°C, 1300 rpm) with 20 μL of 50 mM ammonium formate pH 4.8 buffer and 60 μL of mutanolysin (1 mg.mL^-1^ stock dissolved in 5 mM ammonium formate, and 10 mM ammonium chloride pH 4.8 buffer). Sample was boiled for 10 min at 100°C, cooled down and centrifuged (14000 rpm, 10 min). Supernatant was recovered, dried in vacuo, and resuspended in 25 μL of bidistilled water and 25 μL of 0.5 M ammonium buffer pH 9.0 (adjusted with formic acid). Sample reduction was performed by adding few crystals of tetramethyl ammonium borohydride (30 min, room temperature). Sample pH was adjusted to 3.5-4.0 using 5% formic acid. 20 μL of sample were diluted with 80 μL of 0.2% formic acid (aq), and 10 μl were injected for analysis.

LC–MS/MS setup consisted of an UPLC (Waters Acquity iClass) system and an ESI-QTOF (Bruker Maxis II). Chromatographic separation utilized a Acquity TM Premier BEH C18 (1,7 μm 2,1 x 100 mm) column, mobile phase A (0.1% formic acid (aq)), and mobile phase B (acetonitrile containing 0.1% formic acid). Flow rate was set at 250 μL/min, a column temperature of 30°C, autosampler temperature of 10°C, and the gradient program consisted of 0 to 4% B in 10 min, 4 to 5% B in 20 min, 5 to 10% B in 23 min, 10 to 50% B in 5 min, 50 to 85% B in 4 min and 85 to 0% B in 3 min, plus 10 min re-equilibration at 0 % B (run time 75 min). Mass spectrometry (MS) analysis was performed using the electrospray technique in positive ion detection mode, within a mass range of 50 to 3000 m/z (3.5 kV spray voltage, 0.3 Bar nebulizer, 8.0 L/min dry gas, 300°C dry temperature). In addition to full MS acquisition sequential untargeted fragmentation (Auto MS/MS) were conducted for three precursors per cycle, using variable collision energy based on the precursor mass, all at an acquisition frequency of 10 Hz.

#### *V. spinosum* cell labelling with the fluorescent d-amino acid HADA and confocal microscopy

To evaluate localization of PG incorporation, a 3-day old pre inoculum of *V. spinosum* was diluted and grown to exponential growth phase (OD_600_ 0.4) in modified M13 medium at 30°C. 500 μL of culture was labelled with 250 μM HADA for 2.5 hours at 30°C in the dark with agitation. Cells were washed two times with PBS 1X and spotted on a pad of 1% agarose in PBS, mounted on a microscope Axio Observer7 confocal microscope (Zeiss) equipped with a CSU-W1 spinning disk module (Yokogawa). Images were taken using a Zeiss α Plan-Apochromat 100x/1.46 Oil DIC M27 objective lens and a Prime 95B CMOS camera (Teledyne Photometrics, pixel size: 0.1099 μm), controlled by the SlideBook 6 software. HADA was excited at 405 nm and collected with a 445/45 filter with an exposure time of 100 ms. For each image, a bright-field image was equally collected. Microscopy images processing was performed using Fiji software v1.54f.^55^

#### Scanning and transmission electron microscopy

For TEM and SEM of *V. spinosum*, cells were grown in modified M13 liquid medium at 30°C, collected by centrifugation at three different growth states: early exponential (OD_600_ 0.24), mid-late exponential (OD_600_ 0.74), and stationary (OD_600_ 1.4) phase. For sacculi extraction, *V. spinosum* cells were grown in modified M13 liquid medium (500 mL) at 30°C, collected by centrifugation in late exponential (OD_600_ 0.86), cell pellet was resuspended in 7.5 mL of cold double distilled water and lysed by adding the resuspended pellet, dropwise, to 7.5 mL of boiling 8% SDS within 30 min under vigorous stirring. A second boiled took place for an additional 30 min each, sample was cooled to room temperature and kept at 4°C. Cell pellets preparations were firstly washed in phosphate buffer and later re-suspended, fixed in glutaraldehyde 2.5% and incubated room temperature for 2 hours, followed by three wash steps with cacodylate 0.1 M buffer. For TEM, cells were embedded in agarose and post-fixed with 1% osmium tetroxide for 1 hour and 2% uranyl acetate for 2 hours. Dehydration was carried out dehydrated in a gradual acetone series up to 100% acetone. The samples were then embedded in pure Spurr resin and polymerised at 70°C for 7 hours. Ultrathin sections, approximately 70 nm thick, were prepared using a Leica UC7 ultramicrotome and mounted onto copper grids. The samples were subsequently examined using a Zeiss Libra 120 transmission electron microscope at 120 kV. For SEM, the samples were embedded in agarose blocks, post-fixed with 1% osmium tetroxide for 1 hour, and washed 3 times in 0.1 M cacodylate buffer. They were dehydrated through a gradual acetone series up to 100% acetone and then dried using the critical point drying technique in a Leica CPD300. The samples were coated with Au/Pd (gold/palladium) in a Leica AC600 sputter coater and observed using a high-resolution Zeiss Crossbeam550 scanning electron microscope at 2.0 kV. For visualisation of *V. spinosum* PG 20 μL of the sacculi suspensions were dropped onto a piece of Parafilm. Formvar/carbon-coated copper grids (300 mesh) were placed on top of the drops for 10 min. After incubation, excess liquid was carefully removed from the edges using filter paper. Subsequently, the grids were placed onto a drop of 2% uranyl acetate for 1 min. Excess stain was removed with filter paper, and the grids were left to air dry. Samples were examined using a Zeiss Libra 120 transmission electron microscope operated at 80 kV.

### Quantification and statistical analysis

GraphPad Prism, R and python were used for graphical representation of data and statistical analyses, as described in methods details. The specific statistical tests and cut-offs values used for each experiment are described in the corresponding method details section. Information on replica for each experiment is provided in the corresponding method details section and in the figure legends.

## Supplemental information

Document S1. Figures S1–S9 and Tables S1–S4

Data S1. Excel file containing OD600 values used for graphical representation of Supplementary Figure 1A–1C

Data S2. Excel file containing OD600 values used for graphical representation of Supplementary Figure 1D

Data S3. Excel file containing OD600 values used for graphical representation of Supplementary Figure 1E

Data S4. Excel file containing genome location of unique transposon insertion events in.bed format

Data S5. Excel file containing list of genes classified as essential, not essential or unclear by TraDIS and sliding window method

Data S6. Excel file containing genes with “domain” essential

Data S7. Excel file containing orthologs with *E. coli*

Data S8. Excel file containing orthologs with *P. limnophila*

Data S9. Excel file containing list of organisms used in the phyletic profile of DCW cluster proteins

Data S10. Excel file containing identified ions and obtained masses from LC-MS analysis in V. spinosum peptidoglycan
