# Supplementary material for: Verrucomicrobium spinosum essential genome and the divergence of cell division in the PVC superphylum
Source: iScience. 2025 Jun 30;28(8):113037. doi: 10.1016/j.isci.2025.113037 (PMC12283558; doi:10.1016/j.isci.2025.113037)
Supplement: Document S1. Figures S1–S9 and Tables S1–S4 [file mmc1.pdf]

iScience, Volume *28*

Supplemental information

Verrucomicrobium spinosum essential genome and the divergence of cell division in the PVC superphylum

Valentina Henriques, David Moyano-Palazuelo, Maria Teresa Alonso-Pascual, Manuel Pazos, Damien P. Devos, and Elena Rivas-Marin

Supplemental Figures

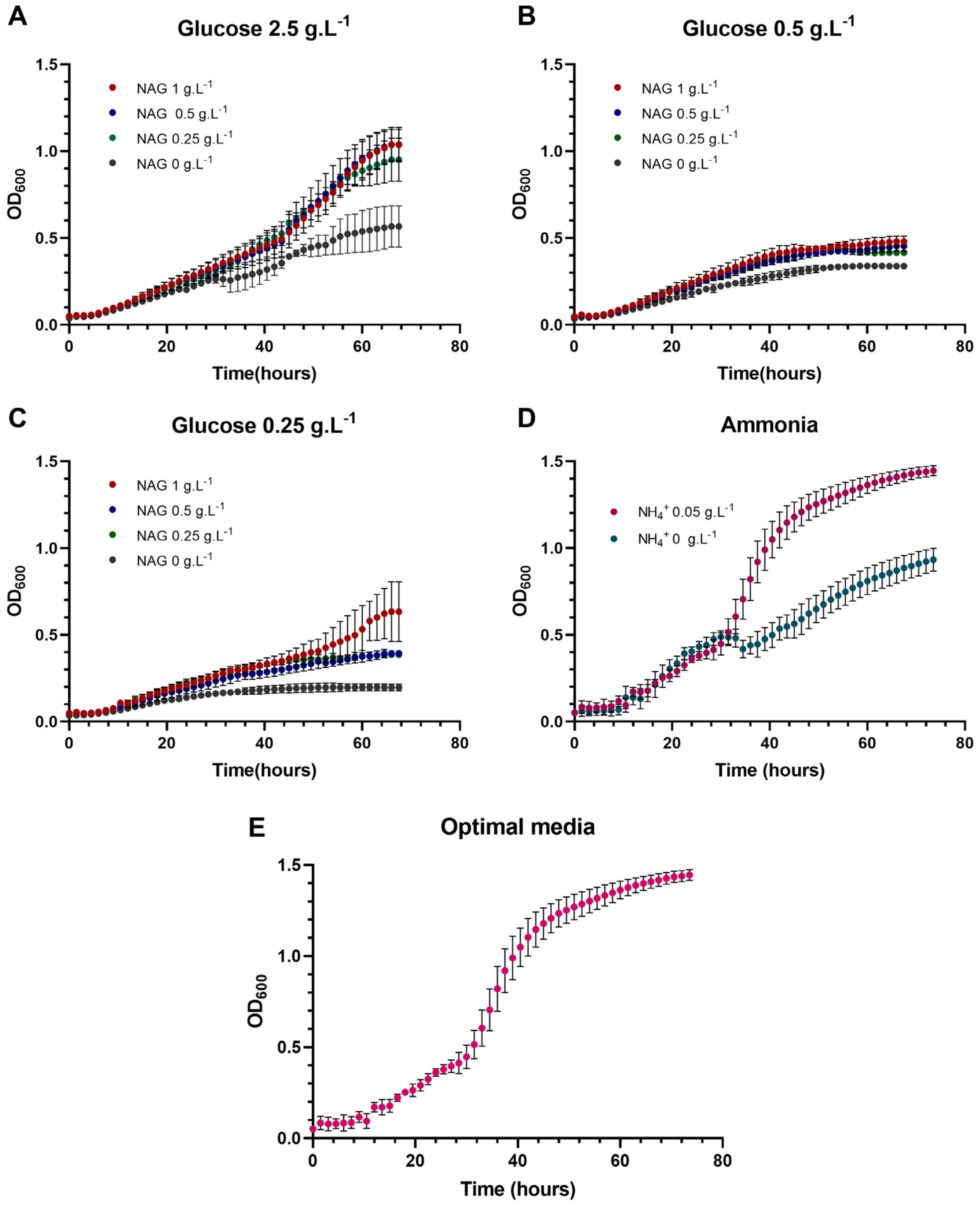

Figure S1. Growth curves of *V. spinosum* under different media. Growth curves were conducted in DSMZ medium 607 (M13) with N-acetylglucosamine (NAG), glucose, and ammonium chloride (ammonia) added at varying concentrations to evaluate their impact on growth. **A–C)** Effect of glucose (2.5, 0.5, and 0.25 $g.L^{-1}$, respectively) and NAG (1, 0.5, 0.25, and 0 $g.L^{-1}$, respectively) concentrations in the absence of ammonia. **D)** Effect of ammonia to the medium supplemented with 1 $g.L^{-1}$ NAG and 2.5 $g.L^{-1}$ glucose. **E)** Growth curve of *V. spinosum* in improved M13 media. The growth rate in the improved medium is characterised by two distinct exponential phases: an initial slower phase (10 h^{-1}) followed by a faster phase (7 h^{-1}). Data points are mean ± standard deviation (S.D.) for *n* = 2 biological replicates in panels A-C and *n* = 5 in panel D and E taken using a Tecan Spark® multimode microplate reader. Individual replica OD_{600} shown in Data S1-3.

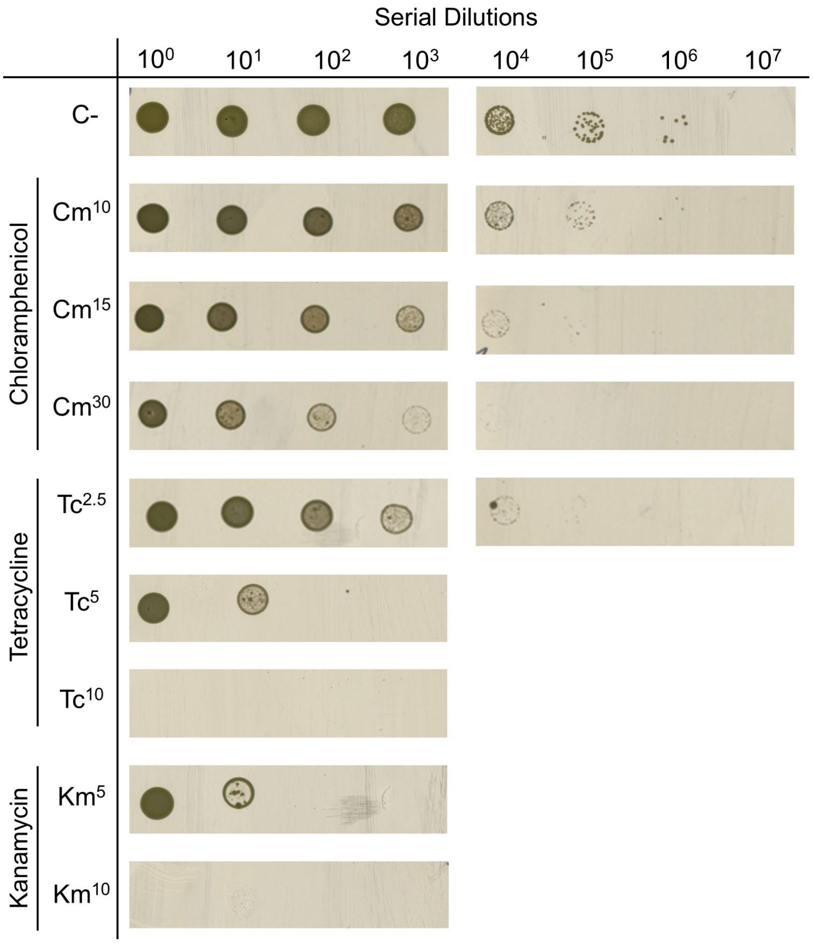

Figure S2. Antimicrobial assay of *V. spinosum*. Serial dilutions of *V. spinosum* culture were spotted onto solidified media supplemented with various antibiotic concentrations. The figure displays only the antibiotic concentrations where some growth was observed in at least one of the tested conditions. Additional antibiotics including ampicillin (25, 50, 100, 200 µg.mL^{-1}), gentamicin (5, 10, 20 µg.mL^{-1}) and streptomycin (25, 50, 100 µg.mL^{-1}) did not support growth at any concentration.

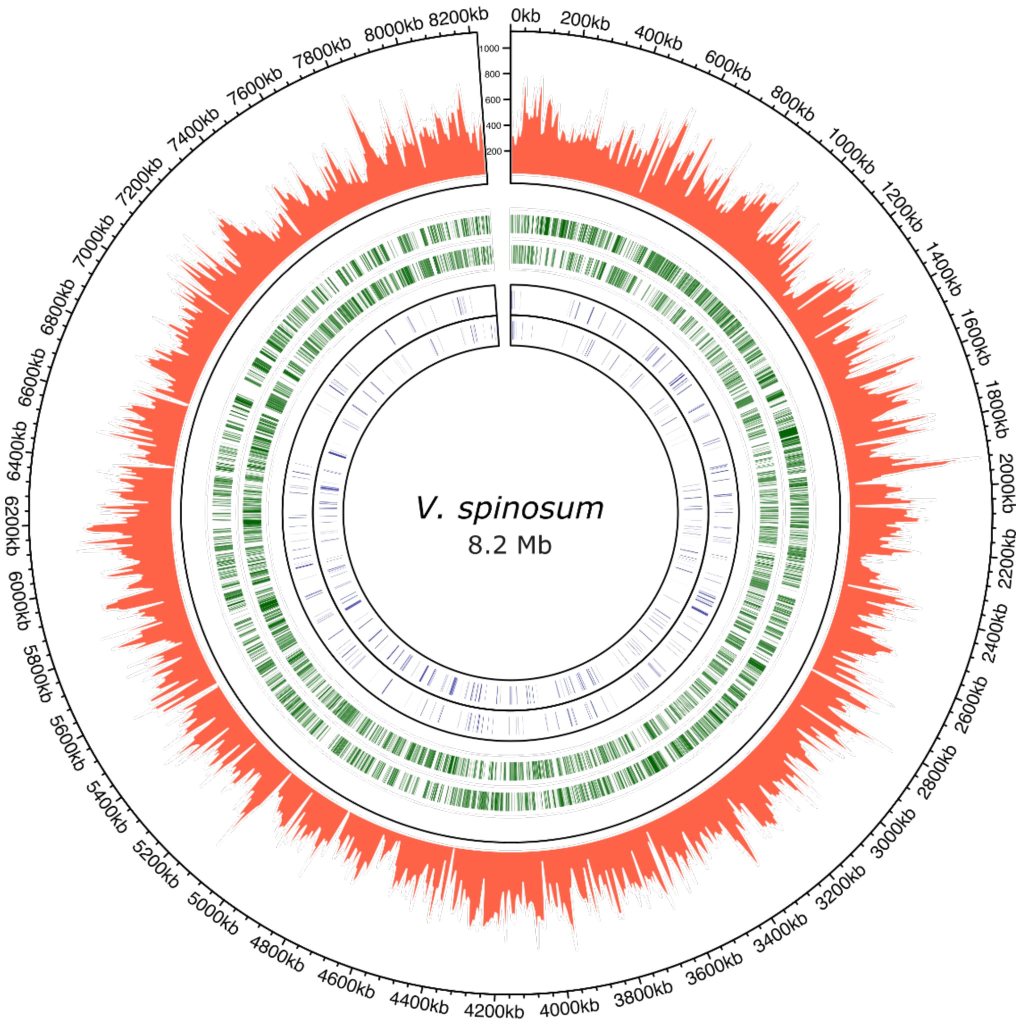

Figure S3. Genome transposon insertion sites of the mutant library. The outermost track marks the genome in base pairs starting at the replication origin. The next inner tracks represent the location and frequency of insertion sequences in the genome (orange). The four innermost circles correspond to sense and anti-sense CDS (green), and the essential genes identified by TraDIS (blue).

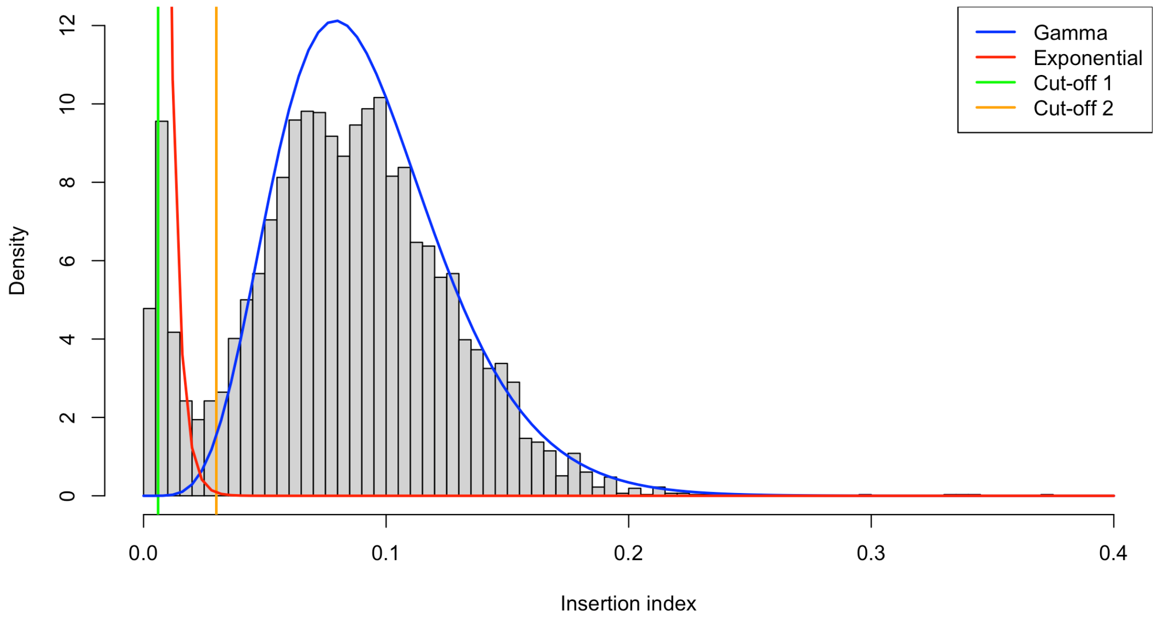

Figure S4. Insertion index distribution in *V. spinosum*. The red curve represents the fit of the left part of this distribution to an exponential distribution. The blue curve represents the fit of the right-hand side to a gamma distribution. The green and orange lines represent the cut-offs used for the fits of the distributions. The histogram and curves represent probability densities, with the histogram normalized such that the total area under the bars is 1.

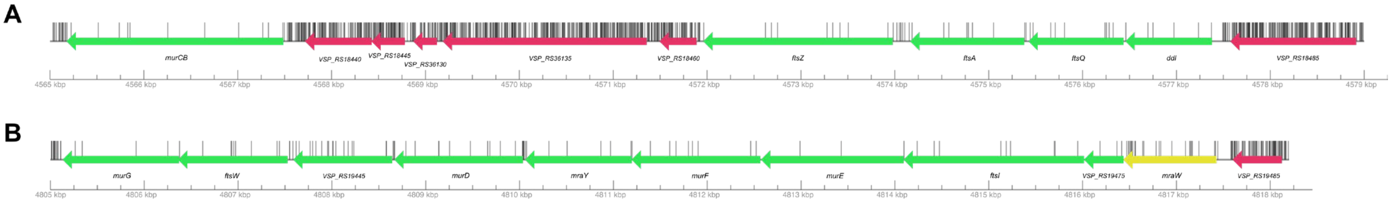

Figure S5. Synteny of *dcw* genes in *V. spinosum*. A) *ftsQAZ* cluster: This region includes division and cell wall-related genes such as *ddl* and *murCB*. The *ftsQAZ* genes are separated from *murCB* by non-division-related genes. **B)** *mraW* cluster: This region contains a cluster of cell division and cell wall synthesis genes, including *ftsI*, *murE*, *murF*, *mraY*, *murD*, *ftsW*, and *murG*. The x-axis shows genomic coordinates in kb. Vertical lines indicate transposon insertion sites. Genes are represented by arrows: essential genes in green, non-essential in red, and genes unclear in yellow.

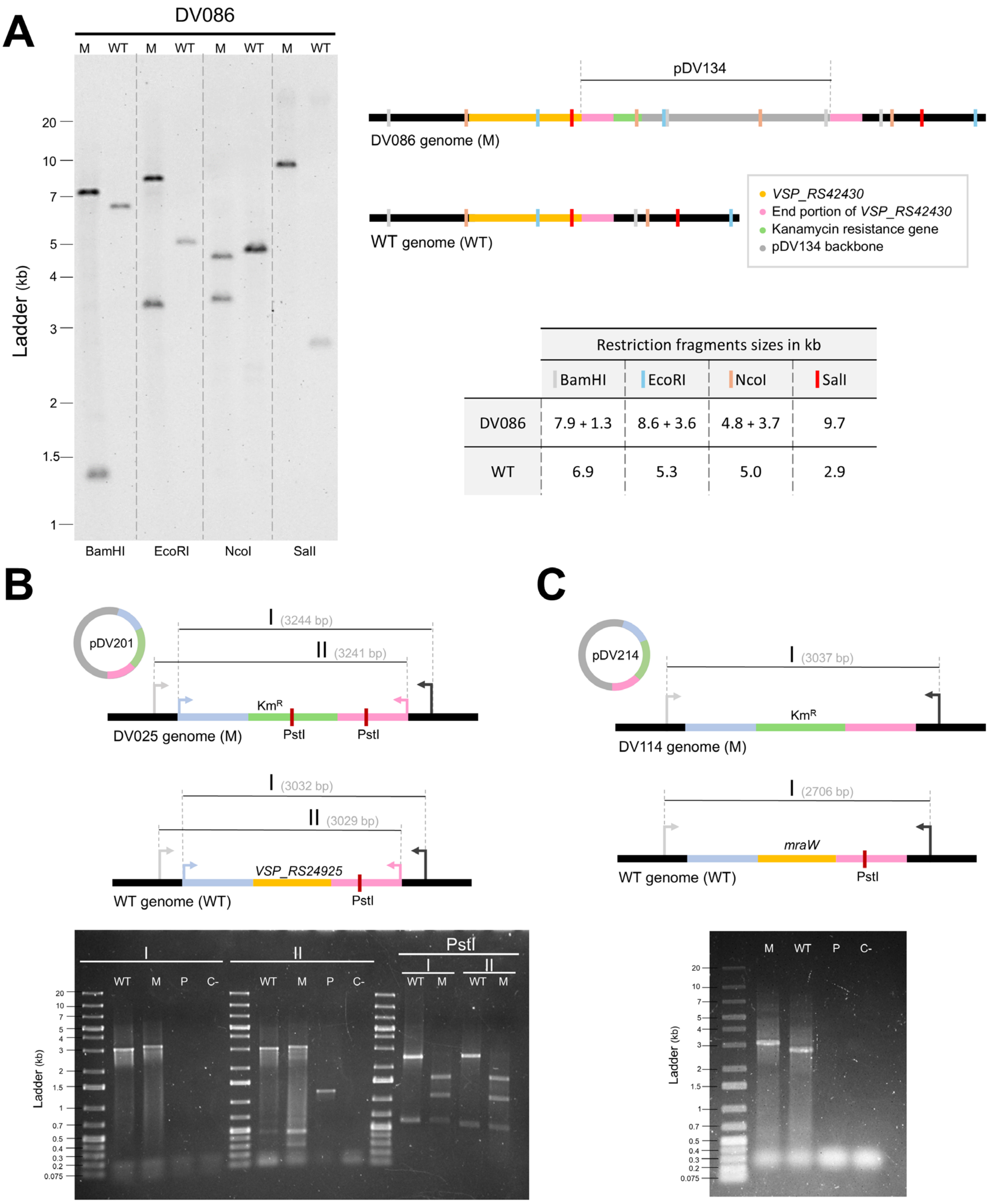

Restriction fragments sizes in kb	BamHI	EcoRI	NcoI	SalI
DV086	7.9 + 1.3	8.6 + 3.6	4.8 + 3.7	9.7
WT	6.9	5.3	5.0	2.9

Figure S6. *V. spinosum* mutants' confirmation. A) Southern blotting confirmation of DV086 insertion mutant: Genomic DNA from *V. spinosum* wild-type (WT) strain and DV086 mutant (M) were digested respectively with BamHI, EcoRI, NcoI and SalI and runed alongside each other. To the right, there is a schematic representation of the restriction enzyme target locations, along with a table showing the expected sizes of the digested fragments. The downstream region of the *VSP_RS42430* gene was used as a probe. **B)** PCR confirmation of DV025 deletion (Δ*VSP_RS24925)* using two distinct primer pairs (**I** and **II**) and subsequent restriction enzyme digestion with respective schematic representation. For the first PCR (**I**), the primers LFR_Whi_fwd (internal primer represented by a blue arrow) and Out DV025_rv (external primer represented by a black arrow) were used to amplify a region of 3032 bp in the WT (lane 2-WT) and 3244 bp in the DV025 mutant (lane 3-M). In the second PCR (**II**), the primers RFR_Whi_rv (internal primer represented by a pink arrow) and Out DV025_fw (external primer

represented by a grey arrow) amplified a region of 3029 bp in the WT (lane 7-WT) and 3241 bp in the mutant (lane 8-M). pDV201 plasmid (P) and negative control (C-) of respective PCR are in lanes 4-5 and 9-10. Digestion of PCR products with Pst**I** are also schematically shown in red. This digestion yielded two fragments in the WT sample (2445 bp and 587 bp for first PCR and 2481 bp and 548 bp for the second), lanes 12 and 14, and three fragments in the DV025 mutant (1603 bp, 1054 bp and 587 bp for first PCR and 1639 bp, 1054 bp and 548 bp for the second) lanes 13 and 15**. C)** DV114 mutant PCR confirmation using the external primers Out DV114 fw and Out DV114 rv to amplify a region of 3037 bp in the mutant (lane 2-M), and 2706 bp in the WT (lane 3-WT), with respective plasmid pDV214 (P) and negative control (C-), schematic representation above the electrophoresis gel.

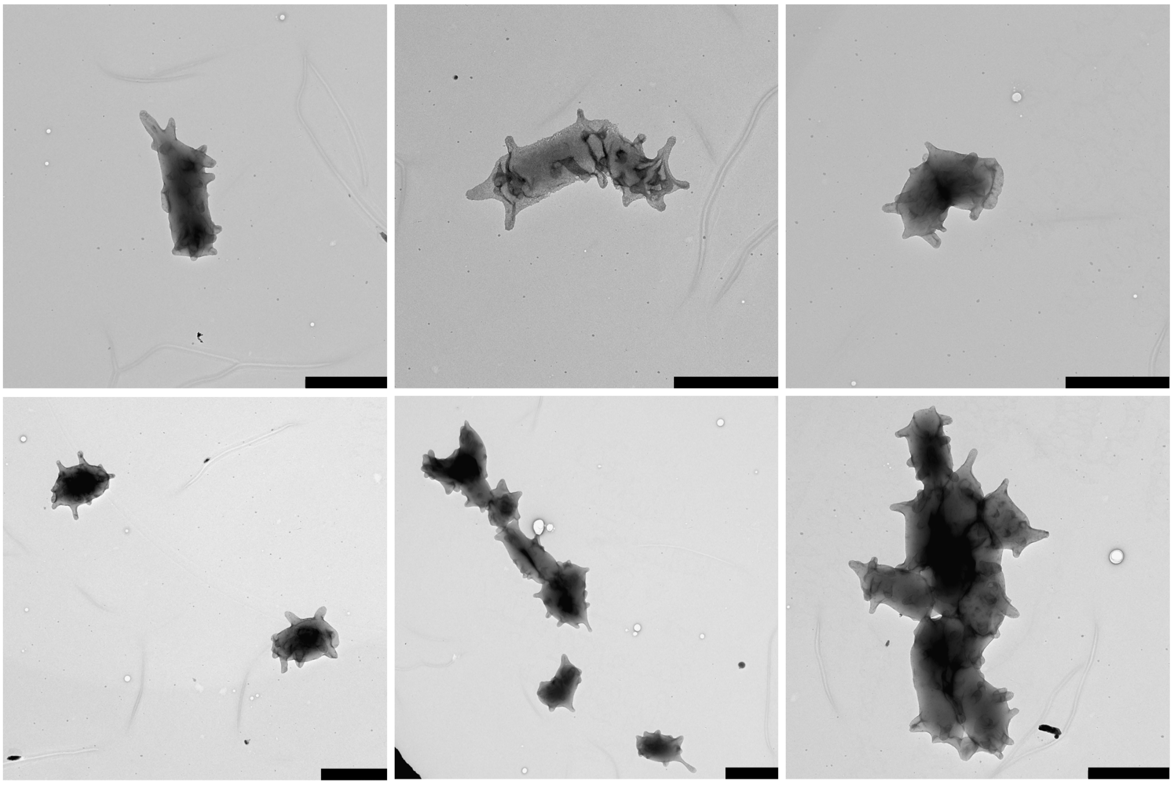

Figure S7. TEM of negative staining of *V. spinosum* sacculi. Scale bars: 2 µm.

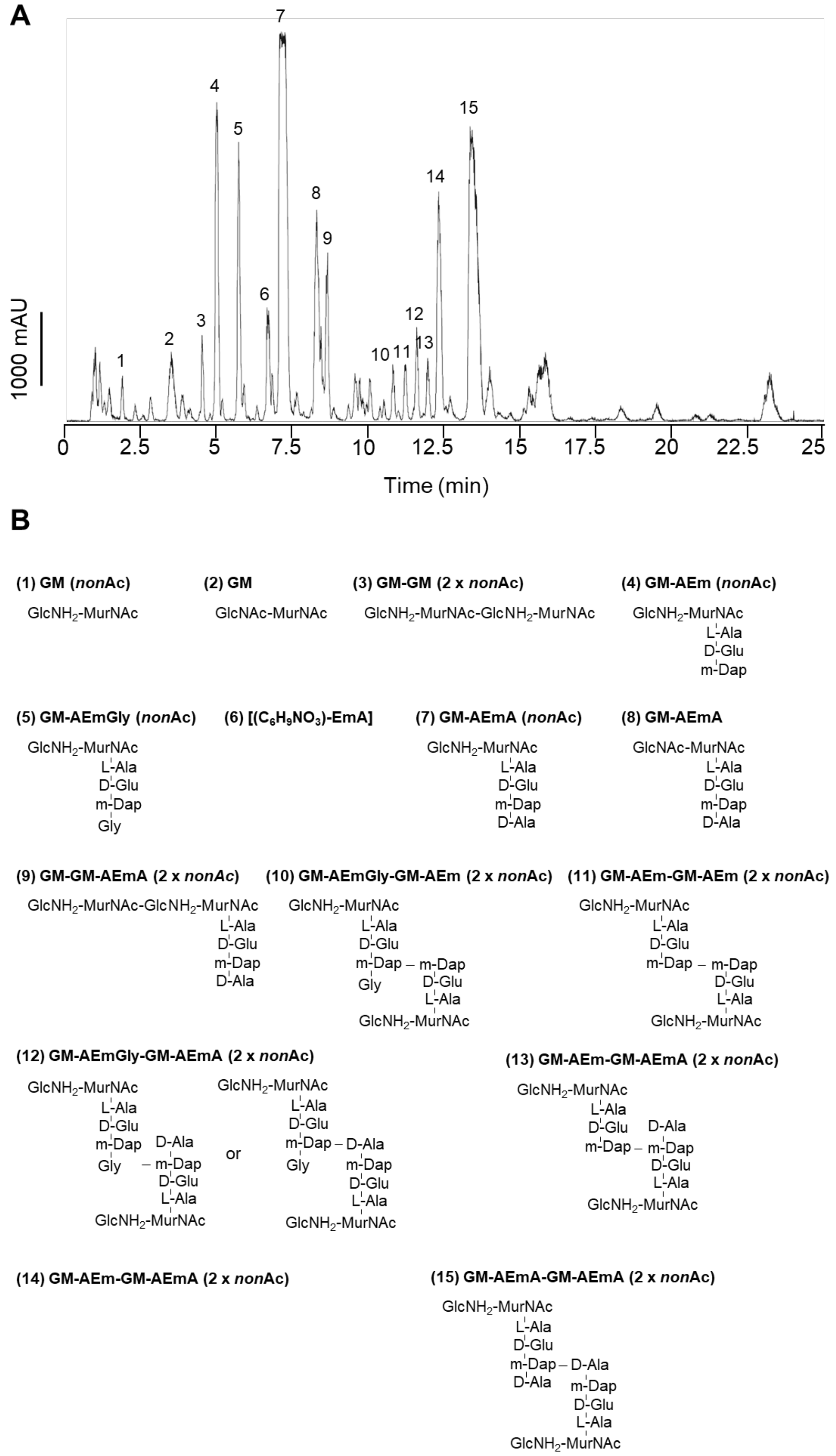

Figure S8. **Muropeptides from *V. spinosum* peptidoglycan. A-** LC-MS analysis. **B-** Proposed structures of the major muropeptides identified. See Table S4 for interpretation of the identified peaks, Figure S9 for the MS and MS/MS fragmentation mass spectra for each muropeptide, and Data S10 for the identified ions and the obtained masses. G, N-acetylglucosamine; M, N-acetylmuramic acid; nonAc, non-acetylated; A, l-Alanine or d-Alanine; E, d-isoglutamate; m, meso-diaminopimelic acid; Gly, glycine.

#1

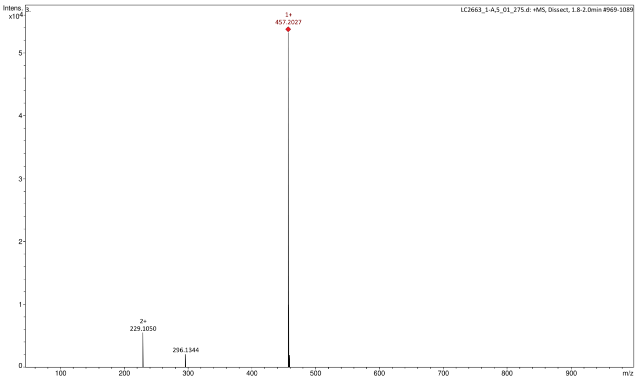

#1

MS/MS 457.2027 (1+)

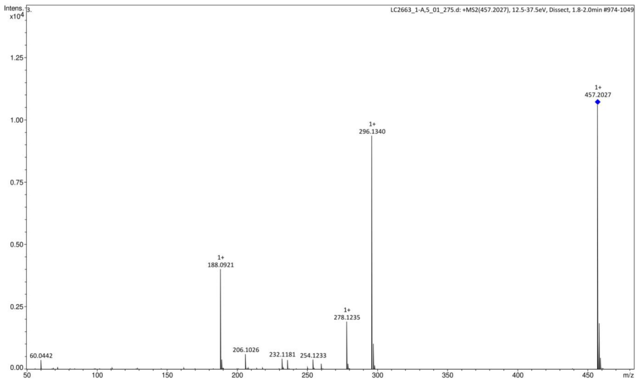

#2

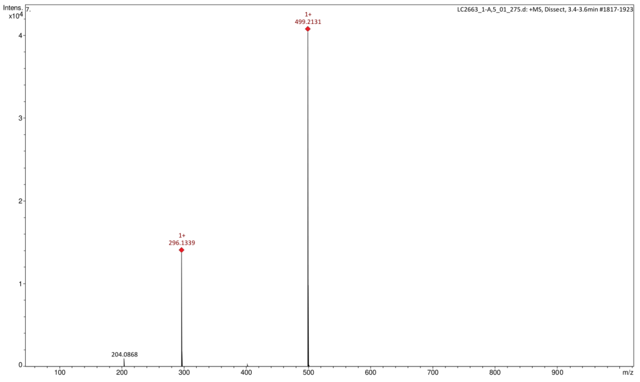

#2

MS/MS 499.2131 (1+)

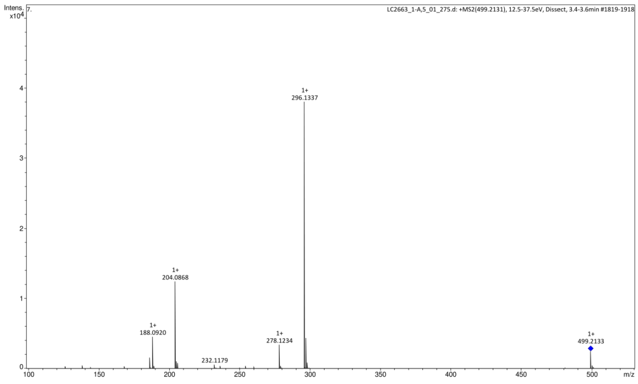

#3

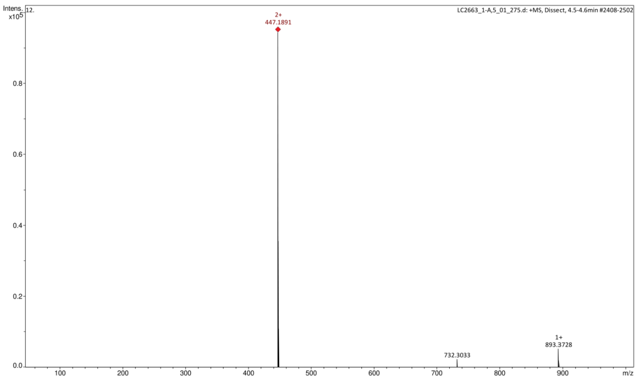

#3

MS/MS 477.1891 (2+)

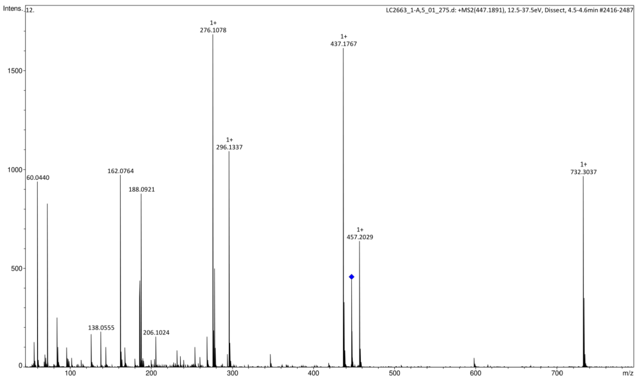

#4

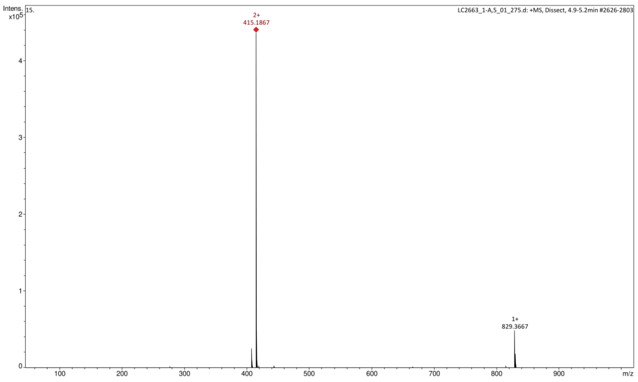

#4

MS/MS 415.1867 (2+)

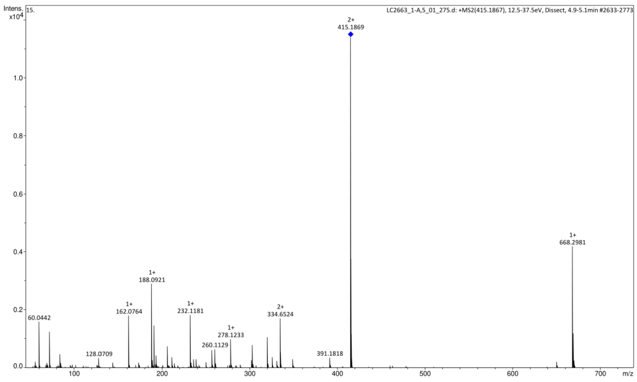

#5

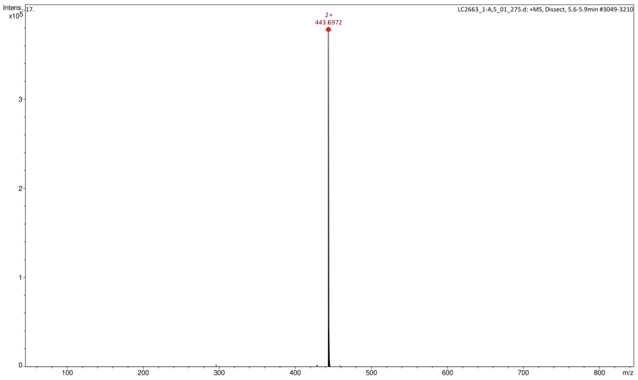

#5

MS/MS 443.6972 (2+)

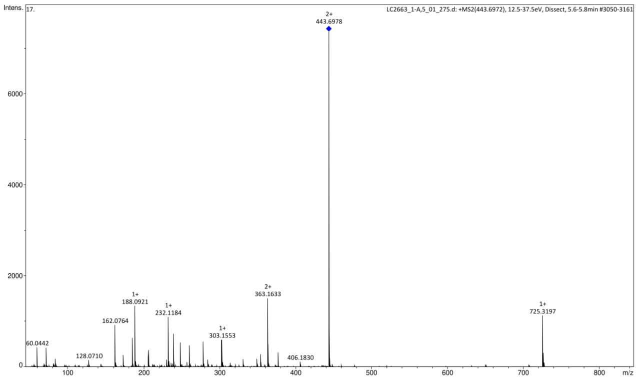

#6

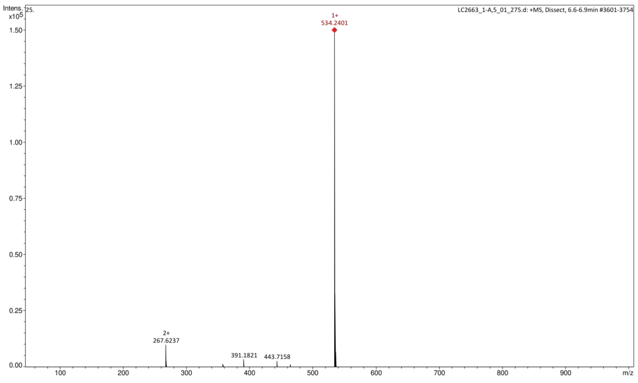

#6

MS/MS 534.2401 (1+)

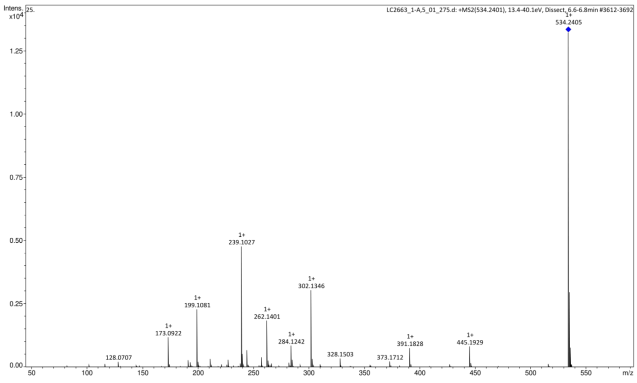

#7

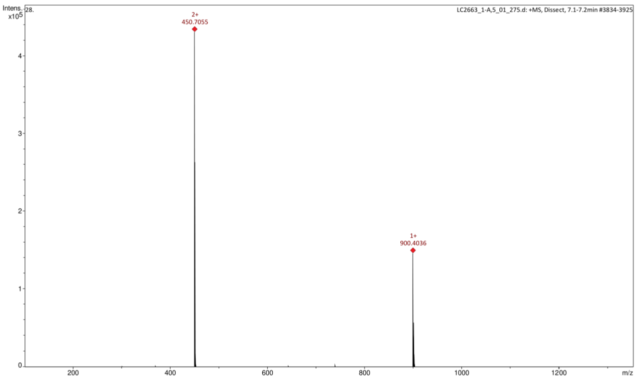

#7

MS/MS 900.4036 (1+)

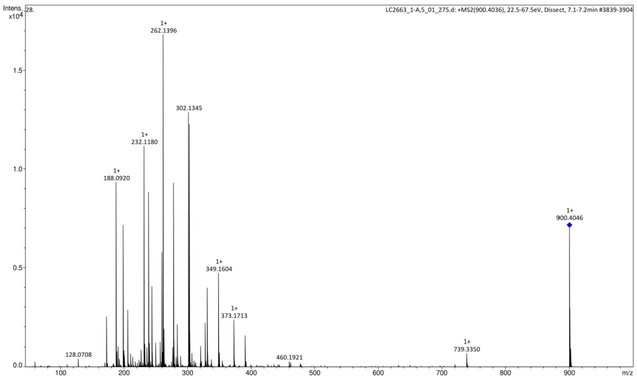

#7

MS/MS 450.7055 (2+)

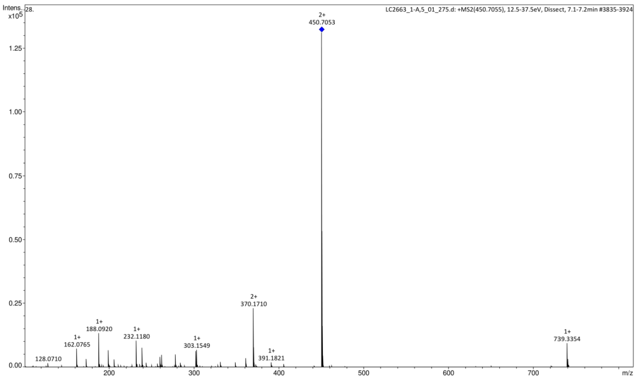

#8

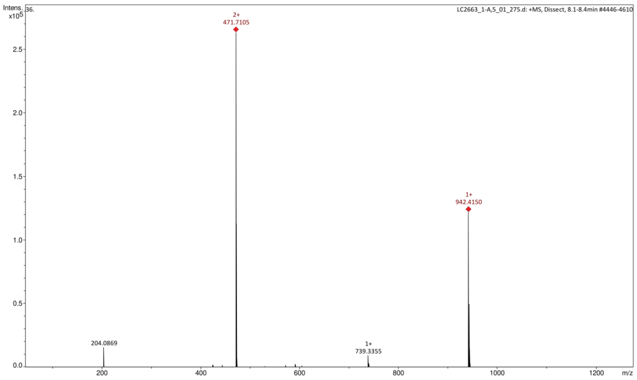

#8

MS/MS 471.7105 (2+)

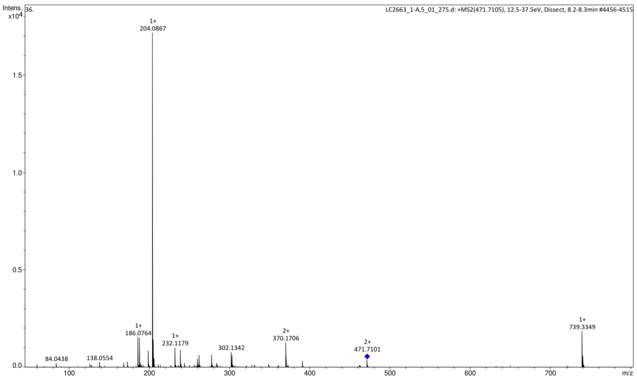

#8

MS/MS 942.4150 (1+)

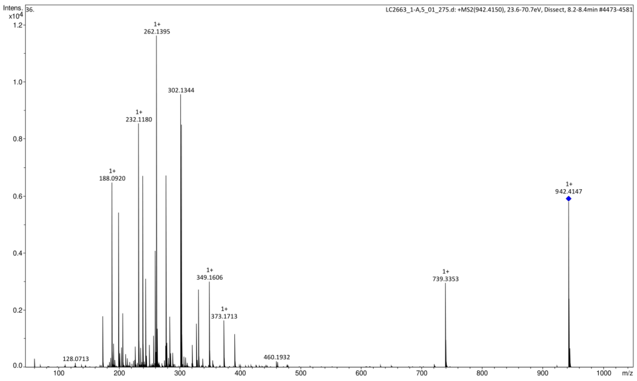

#9

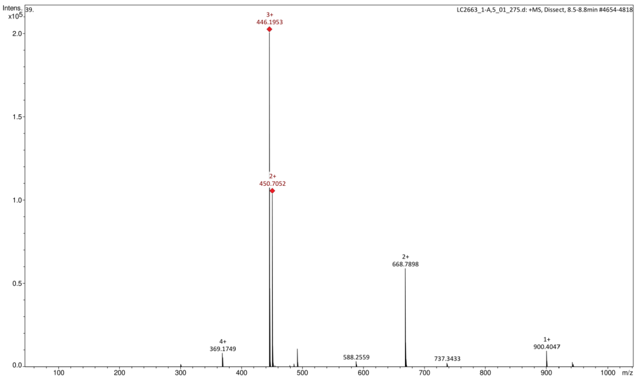

#9

MS/MS 450.7052 (2+)

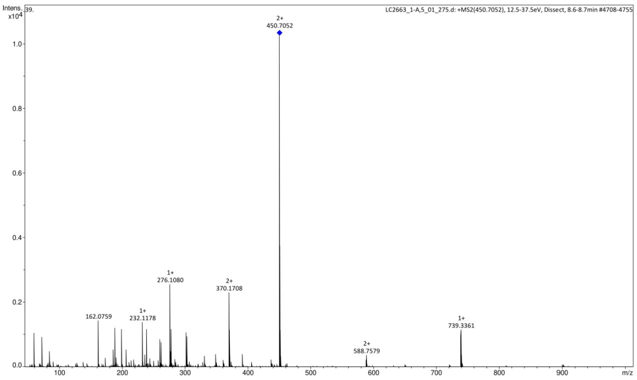

#9

MS/MS 446.1953 (3+)

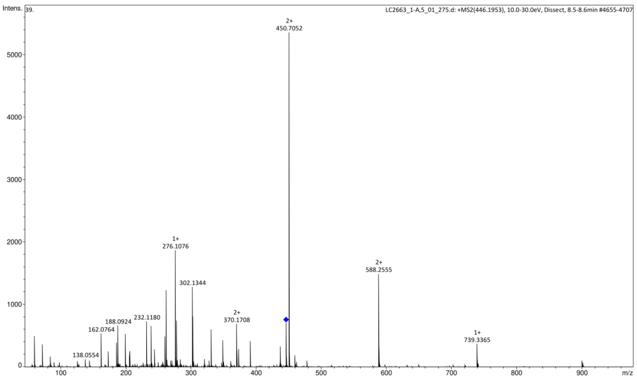

#10

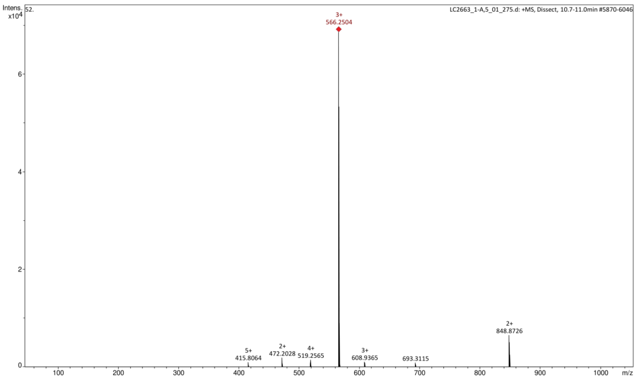

#10

MS/MS 566.2504 (3+)

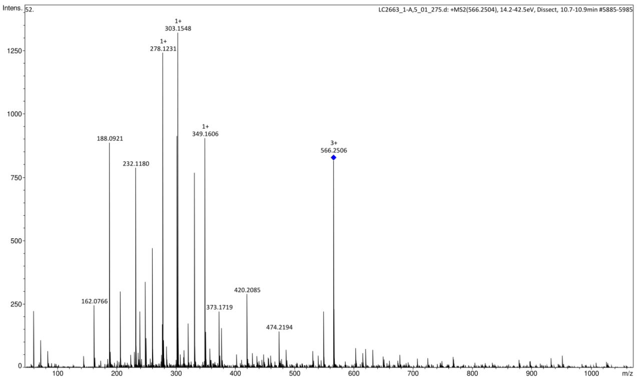

#11

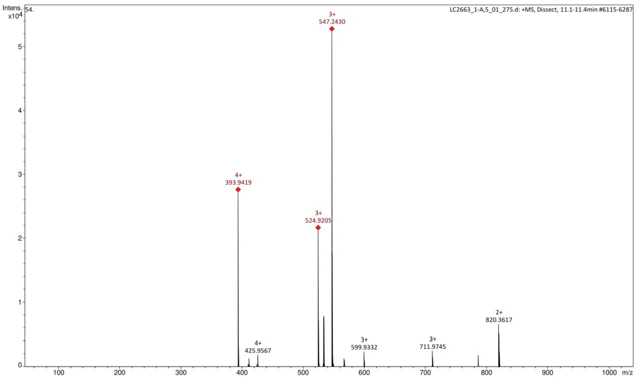

#11

MS/MS 524.9205 (3+)

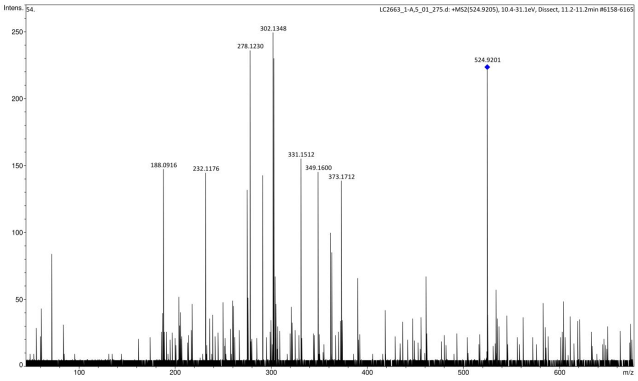

#11

MS/MS 547.2430 (3+)

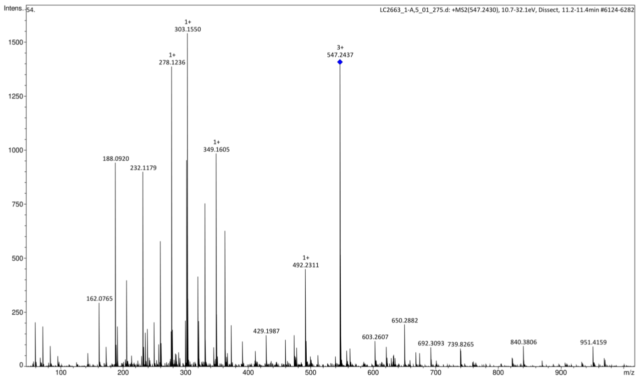

#11

MS/MS 393.9419 (4+)

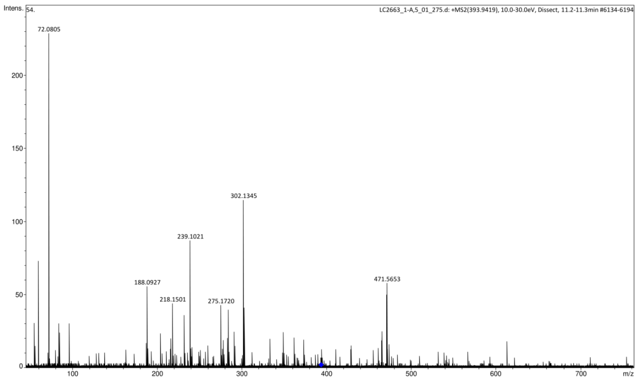

#12

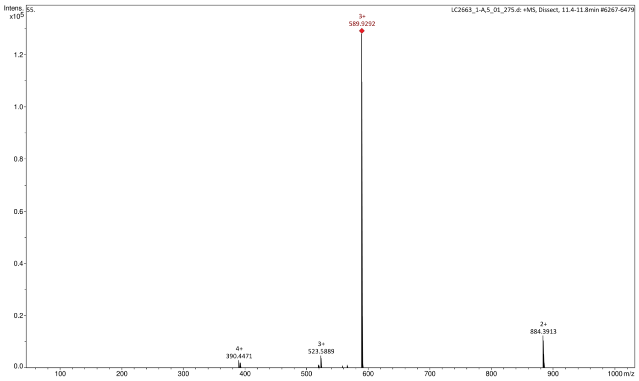

#12

MS/MS 589.9292 (3+)

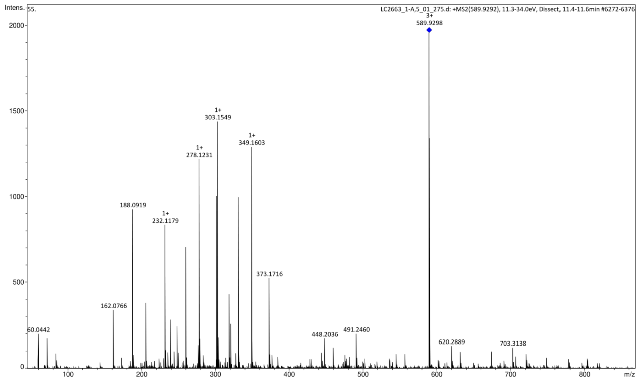

#13

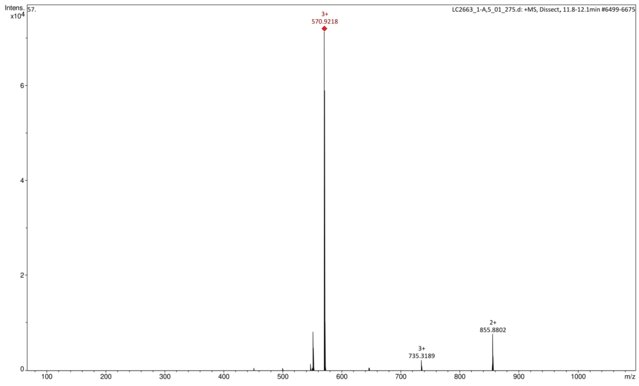

#13

MS/MS 570.9218 (3+)

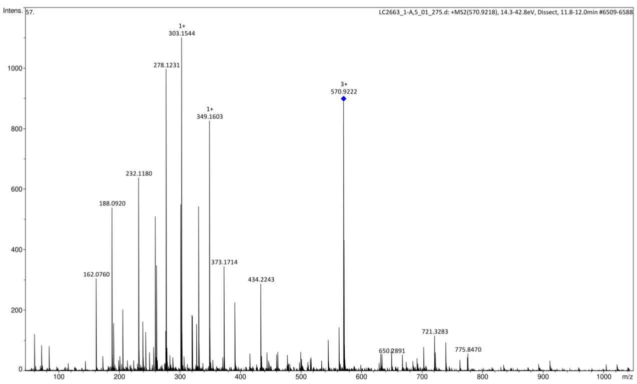

#14

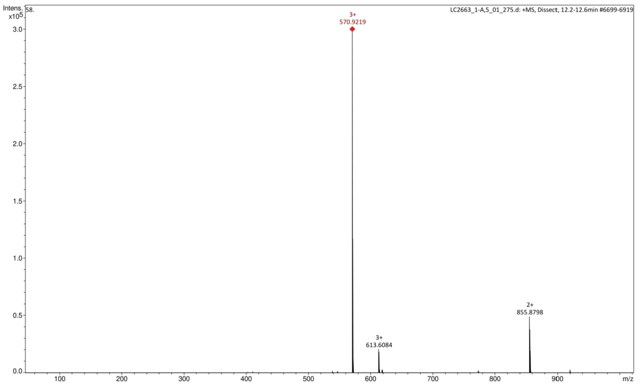

#14

MS/MS 570.9219 (3+)

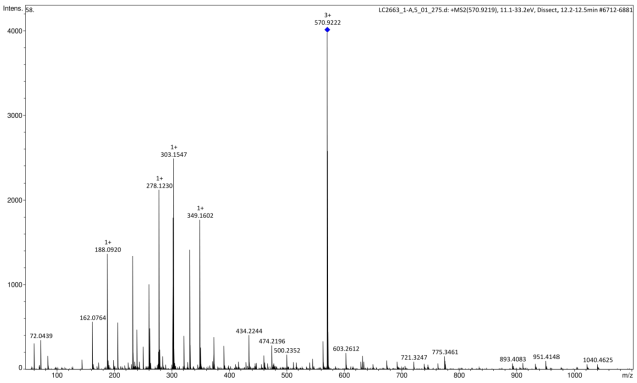

#15

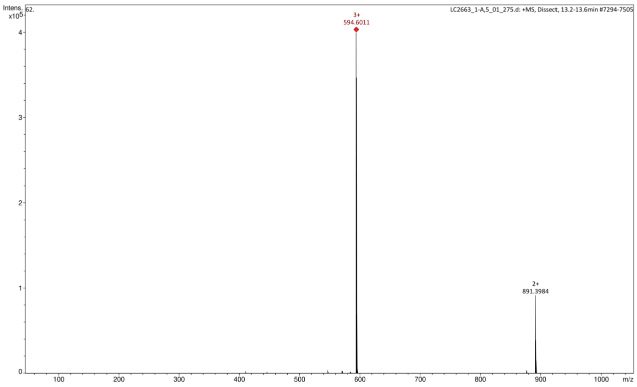

#15

MS/MS 594.6011 (3+)

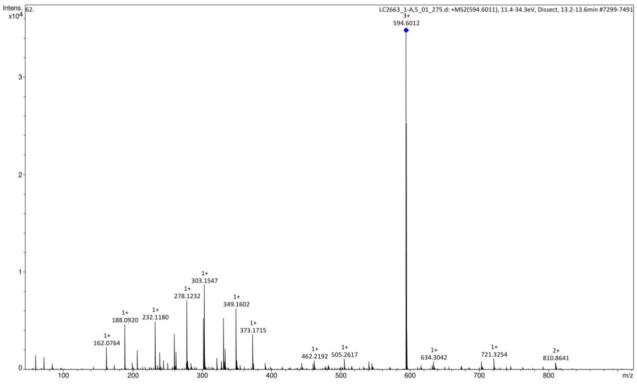

Figure S9. MS and MS/MS fragmentation mass spectra for each *V. spinosum* muropeptide identified. Peak number correspond to the assigned ones in Figure S6 and are indicated by the symbol "#". Each panel displays the fragmentation pattern of the corresponding peak along with its respective charges.

Supplemental Tables

Table S1. Strains used in this work.

Strain name	Genotype	Reference
Escherichia coli DH5α	F^- φ80*lacZ*ΔM15 Δ(*lacZYA-argF)U169 recA1 endA1hsdR17*(r_K^- m_K^-) *supE44 thi-1 gyrA relA1*	(Hanahan[1])
E. coli ER2566	*fhuA2 lacZ::T7 gene1 [lon] ompT gal sulA11 R(mcr-73::miniTn10*--Tet^S*)2 [dcm] R(zgb-210::Tn10*--Tet^S*) endA1 Δ(mcrC-mrr)114::IS10*	New England Biolab
Verrucomicrobium spinosum DSM 4136	Wild-type strain	(Schlesner[2])
V. spinosum DV086	*VSP_RS42430*::pDV134	This work
V. spinosum DV025	Δ*VSP_RS24925*. Km^R	This work
V. spinosum DV085	*VSP_RS24925*'::Tn*5*.Km^R	This work
V. spinosum DV114	Δ*mraW*. Km^R	This work

Table S2. Plasmids used in this work.

Plasmid name	Main features source	Source
pTXB1-Tn*5*	IPTG-inducible expression of Tn*5* transposase fused to Mxe Intein and Chitin-binding domain	Addgene plasmid 60240
pMPO1012	Mobilizable, ColE1, Km^R, containing the *mut3a-gfp* expressed under a heterologous promoter.	(Rivas-Marin et al.[3])
pUTminiTn*5*km	Ap^R; R6K, Tn*5*-based delivery plasmid with Km^R	(Herrero et al.[4])
pEX18Tc	Tc^R; *oriT*$^+$ *sacB*$^+$, gene replacement vector with MCS from pUC18	(Hoang et al.[5])
pDV134	907 bp fragment of *VSP_RS42430* gene from *V. spinosum* in pMPO1012. Km^R	This work
pDV201	968 bp upstream and 960 bp downstream of *VSP_RS24925* gene from *V. spinosum* flanking a kanamycin resistance gene cloned into pEX18Tc. Km^R, Tc^R	This work
pDV214	858 bp upstream and 815 bp downstream of *mraW* gene from *V. spinosum* flanking a kanamycin resistance gene cloned into pEX18Tc. Km^R, Tc^R	This work

Table S3. Oligonucleotides used in this work. Underlined restriction sites.

Primer name	Primer sequence	Target
Vs_RFR_fw_endo	TACAAGCTTTAGGATCCCAGATTGGTCATTACTCGC	*VSP_RS42430* downstream region
Vs_RFR_rv_endo	TACAAGCTTGAGGCTAGTTCGATTCTGG	*VSP_RS42430* downstream region
LFR_Whi_fwd	TTAAAGCTTGCAGAGATTCCCATTGTGCTC	*VSP_RS24925* upstream region
LFR_Whi_rv	ACTGGATCCTGAAGACGGGGACATGGAGG	*VSP_RS24925* upstream region
RFR_Whi_fwd	ACTGGATCCCATGAGCCTCTCCAGCCCTG	*VSP_RS24925* downstream region
RFR_Whi_rv	CAGGAATTCTTCTTCCACCATGGCTTCTTTG	*VSP_RS24925* downstream region
LFR_mraW_fw	ATTGAATTCTGTCATGGAGAGACACAAGAC	*mraW* upstream region
LFR_mraW_rv	ATAGGATCCATTGAAACGTCAGTTGGGAGC	*mraW* upstream region
RFR_mraW_fw	TTAGGATCCCGTTCCCGCAGCGCCAAAC	*mraW* downstream region
RFR_mraW_rv	ATTAAGCTTTGCCGTTCACCACTTCAGTG	*mraW* downstream region
Km BamHI fwd	GTTGGATCCGCGTCGGCTTGAACGAATTG	Kanamycin resistant gene
Km BamHI rv	TGAGGATCCCATTTCGAACCCCAGAGTCC	Kanamycin resistant gene
Km IS fwd	*CTGTCTCTTATACACATCT*GCGTCGGCTTGAACGAATTG	Kanamycin resistant gene bearing Tn*5* IS sequence
Km IS rv	*CTGTCTCTTATACACAT*CTCATTTCGAACCCCAGAGTCC	Kanamycin resistant gene bearing Tn*5* IS sequence
Map Tn*5* A fw	ATCAGGACATAGCGTTGGC	Tn*5* transposon
Map Tn*5* B fw	AAGAGCTTGGCGGCGAATG	Tn*5* transposon
Seq nMCP fwd	CTTGAGTTTGTAACAGCTGC	To sequence pDV134
Seq nMCP rv	GACGATGAGCGCATTGTTAG	To sequence pDV134
T3	AATTAACCCTCACTAAAGGG	To sequence pDV201
T7	GTAATACGACTCACTATAGGGC	To sequence pDV201
Out DV025 fwd	TGGACAGCCAATGGTGATGC	To validate by PCR DV025
Out DV025 rv	ATCGCTGGCGATTTCATCTGC	To validate by PCR DV025
Out DV114 fw	TGGTGCAAGATGGGGAAATC	To validate by PCR DV114
Out DV114 rv	TCGCGATACTGGGAAATCAC	To validate by PCR DV114

Table S4. Reduced muropeptides identified from *V. spinosum* peptidoglycan.

Peak[a]	Muropeptide[b]	Observed mass over charge m/z	Calculated neutral mass	Predicted neutral mass
1	GM (nonAc)	457.2027 (1+)	456.1954	456.1955
2	GM	499.2131 (1+)	498.2058	498.2061
3	GM-GM (2 x nonAc)	447.1891 (2+)	892.3637	892.3648
4	GM-AEm (nonAc)	415.1867 (2+)	828.3590	828.3600
5	GM-AEmGly (nonAc)	443.6972 (2+)	885.3798	885.3815
6	$C_6H_9NO_3$-EmA	534.2401 (1+)	533.2328	
7	GM-AEmA (nonAc)	450.7055 (2+) / 900.4036 (1+)	899.3960	899.3971
8	GM-AEmA	471.7105 (2+) 942.4150 (1+)	941.4068	941.4077
9	GM-GM-AEmA (2 x nonAc)	446.1953 (3+)	1335.5641	1335,5664
10	GM-AEmGly-GM-AEm (2 x nonAc)	566.2504 (3+)	1695.7291	1695.731
11	GM-AEm-GM-AEm (2 x nonAc)	547.2430 (3+)	1638.7074	1638,709
12	GM-AEmGly-GM-AEmA (2 x nonAc)	589.9292 (3+)	1766.7657	1766,768
13	GM-AEm-GM-AEmA (2 x nonAc)	570.9218 (3+)	1709.7433	1709,747
14	GM-AEm-GM-AEmA (2 x nonAc)	570.9219 (3+)	1709.7438	1709,747
15	GM-AEmA-GM-AEmA (2 x nonAc)	594.6011 (3+)	1780.7813	1780.784

[a] Peak number correspond to the assigned ones in Figure 6. [b] Nomenclature: G, N-acetylglucosamine; M, N-acetylmuramic acid; nonAc, nonacetylated; A, l-Alanine or d-Alanine; E, d-isoglutamate; m, meso-diaminopimelic acid; Gly, glycine

References

1. Hanahan, D. (1983). Studies on transformation of *Escherichia coli* with plasmids. J. Mol. Biol. *166*, 557–580. https://doi.org/10.1016/S0022-2836(83)80284-8.

2. Schlesner, H. (1987). Verrucomicrobium spinosum gen. nov., sp. nov.: a Fimbriated Prosthecate Bacterium. Syst. Appl. Microbiol. *10*, 54–56. https://doi.org/10.1016/S0723-2020(87)80010-3.

3. Rivas-Marín, E., Canosa, I., Santero, E., and Devos, D.P. (2016). Development of Genetic Tools for the Manipulation of the Planctomycetes. Front. Microbiol. *7*. https://doi.org/10.3389/fmicb.2016.00914.

4. Herrero, M., de Lorenzo, V., and Timmis, K.N. (1990). Transposon vectors containing non-antibiotic resistance selection markers for cloning and stable chromosomal insertion of foreign genes in gram-negative bacteria. J. Bacteriol. *172*, 6557–6567. https://doi.org/10.1128/jb.172.11.6557-6567.1990.

5. Hoang, T.T., Karkhoff-Schweizer, R.R., Kutchma, A.J., and Schweizer, H.P. (1998). A broad-host-range Flp-FRT recombination system for site-specific excision of chromosomally-located DNA sequences: application for isolation of unmarked Pseudomonas aeruginosa mutants. Gene *212*, 77–86. https://doi.org/10.1016/s0378-1119(98)00130-9.
